# Systems Biology Approaches Reveal a Specific Interferon-Inducible Signature in HTLV-1 Associated Myelopathy

**DOI:** 10.1371/journal.ppat.1002480

**Published:** 2012-01-26

**Authors:** Sonja Tattermusch, Jason A. Skinner, Damien Chaussabel, Jacques Banchereau, Matthew P. Berry, Finlay W. McNab, Anne O'Garra, Graham P. Taylor, Charles R. M. Bangham

**Affiliations:** 1 Department of Immunology, Imperial College London, London, United Kingdom; 2 Baylor Institute for Immunology Research-ANRS Center for Human Vaccines, INSERM U8996, Dallas, Texas, United States of America; 3 Benaroya Research Institute, Seattle, Washington, United States of America; 4 Inflammation and Virology DTA, Hoffman-La Roche Inc, Nutley, New Jersey, United States of America; 5 Division of Immunoregulation, Medical Research Council National Institute for Medical Research, London, United Kingdom; 6 Department of Respiratory Medicine, St Mary's Hospital, Imperial College Healthcare NHS Trust, London, United Kingdom; 7 GU Medicine and Communicable Diseases, Imperial College London, London, United Kingdom; University of Pennsylvania School of Medicine, United States of America

## Abstract

Human T-lymphotropic virus type 1 (HTLV-1) is a retrovirus that persists lifelong in the host. In ∼4% of infected people, HTLV-1 causes a chronic disabling neuroinflammatory disease known as HTLV-1-associated myelopathy/tropical spastic paraparesis (HAM/TSP). The pathogenesis of HAM/TSP is unknown and treatment remains ineffective. We used gene expression microarrays followed by flow cytometric and functional assays to investigate global changes in blood transcriptional profiles of HTLV-1-infected and seronegative individuals. We found that perturbations of the p53 signaling pathway were a hallmark of HTLV-1 infection. In contrast, a subset of interferon (IFN)-stimulated genes was over-expressed in patients with HAM/TSP but not in asymptomatic HTLV-1 carriers or patients with the clinically similar disease multiple sclerosis. The IFN-inducible signature was present in all circulating leukocytes and its intensity correlated with the clinical severity of HAM/TSP. Leukocytes from patients with HAM/TSP were primed to respond strongly to stimulation with exogenous IFN. However, while type I IFN suppressed expression of the HTLV-1 structural protein Gag it failed to suppress the highly immunogenic viral transcriptional transactivator Tax. We conclude that over-expression of a subset of IFN-stimulated genes in chronic HTLV-1 infection does not constitute an efficient host response but instead contributes to the development of HAM/TSP.

## Author Summary

Infection with the Human T Lymphotropic virus is widespread in the tropics and subtropics, where it causes a chronic disabling disease of the nervous system abbreviated as HAM/TSP. There is no effective treatment available for HAM/TSP, because it is not understood how the virus causes the neuronal damage that results in the clinical symptoms of weakness and paralysis of the legs. Here, we compared the frequencies of cell populations and gene expression profiles from diseased and asymptomatic HTLV-1 carriers to identify abnormalities in biological pathways that cause HAM/TSP. We discovered a distinct group of genes that is over-expressed in white blood cells in patients with HAM/TSP, but not asymptomatic HTLV-1 carriers or patients with the clinically similar disease multiple sclerosis. The expression of these genes is induced by interferons, a group of anti-viral proteins that are usually beneficial to the host but can also cause inflammation. We also found that interferons did not efficiently suppress HTLV-1 protein expression *in vitro*. We conclude that interferons do not control chronic HTLV-1 infection but instead contribute to the development of HAM/TSP. Our study provides new insights into the development of HTLV-1-associated diseases and opens new areas of therapeutic intervention.

## Introduction

HTLV-1 is an exogenous retrovirus that is widespread in the tropics and subtropics [1], [2]. Chronic infection is characterised by a strong humoral and cellular immune response against the virus [3].

Whilst the majority of HTLV-1 carriers efficiently control the infection and remain clinically asymptomatic lifelong, some 1–4% develop a neurodegenerative inflammatory disorder known as HAM/TSP [1]. Here, mononuclear cell infiltrates in the central nervous system are associated with myelin and axonal destruction [1], [4]. Clinically, patients present with multiple sclerosis-like symptoms including progressive spastic paraparesis, sensory loss and disturbances of bladder or bowel function [1], [5]. The sequence and nature of the events that lead to the observed neuronal damage in HAM/TSP are not known and because of the lack of a suitable animal model a direct investigation of pathogenic mechanisms is not possible. Thus, clinical management of HAM/TSP is unsatisfactory and current treatment remains only palliative.

To date, the strongest correlate of risk of HAM/TSP remains a high HTLV-1 proviral load [6], i.e. the percentage of peripheral blood mononuclear cells that carry the provirus. The proviral load can differ among individuals by 1000-fold; the mean proviral load is ∼15-fold higher in patients with HAM/TSP than in asymptomatic HTLV-1 carriers (ACs) [6]. However, a significant proportion of infected individuals who present with high HTLV-1 proviral loads never develop the inflammatory disease, suggesting that additional factors contribute to the pathogenesis of HAM/TSP.

Although other risk factors have been proposed, few differ systematically between ACs and patients with HAM/TSP after normalisation for proviral load: the frequency of certain lymphocyte subsets (HTLV-1-specific CD4^+^ T cells [7]; FoxP3^+^CD4^+^ T cells [8]; natural killer (NK) cells [9]); the level of expression of HTLV-1 genes [10] and the pattern of integration of the HTLV-1 provirus in the host cell genome [11]. However, the contribution of each of these factors to the pathology of HAM/TSP is not known.

Blood transcriptional profiles have revealed previously unsuspected pathways of pathogenesis in autoimmune and infectious diseases [12], [13]. Exposure to host or pathogen-derived immunogenic factors trigger changes in gene expression in peripheral blood leukocyte populations that reflect the host immune response mounted against pathogens as well as pathologic conditions of the immune system [14]. Thus, patient blood samples provide an easily accessible source of immunophenotypic information and are of particular value in HTLV-1 infection, in which CD4^+^ T cells constitute the major viral reservoir [15]. Previous studies on gene expression in HTLV-1 infection have focused on specific cell subpopulations (HTLV-1-infected cells, CD4^+^ T cells, CD8^+^ T cells) [16]–[21]. In contrast, whole blood studies integrate gene expression profiles of all leukocytes. Thus, the resulting transcriptional profiles provide a comprehensive overview of the status of the immune system and associated pathological changes.

In this study, we aimed to identify the principal biological pathways and cell types that are deregulated in HTLV-1 infection and HAM/TSP by quantifying and analyzing differences in blood gene expression patterns of HTLV-1-infected individuals and seronegative controls. Unexpectedly, although the class I-restricted T cell response plays a pivotal role in controlling viral replication and risk of disease [22], [23], the outstanding differences in the transcriptional patterns lay in IFN-stimulated genes. We found that over-expression of a distinct subset of IFN-stimulated genes – a ‘transcriptional signature’ – was associated with presence of the inflammatory disease and positively correlated with clinical severity of HAM/TSP. This IFN-inducible transcriptional signature was absent in patients with multiple sclerosis, suggesting distinct pathogenetic pathways in the two diseases despite their clinical similarities. We conclude that IFNs do not efficiently control chronic HTLV-1 infection but may instead contribute to HAM/TSP pathogenesis. Our results identify IFN signaling as a key factor and a novel therapeutic target in HAM/TSP.

## Results

### The blood transcriptional signature of HTLV-1 infection

**Figure 1:**
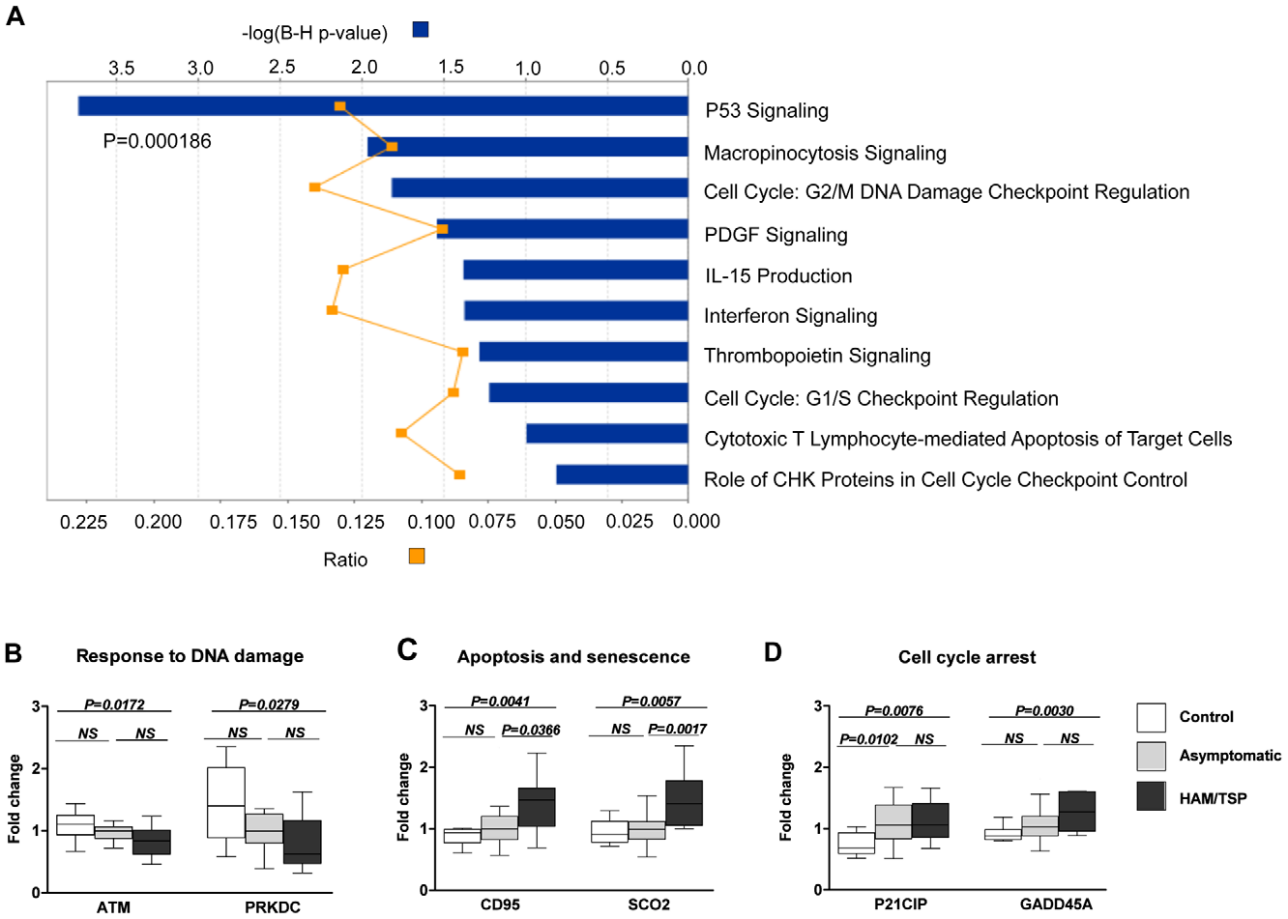
Canonical pathway analysis of the 542-gene blood transcriptional signature of HTLV-1 infection. (A) The biological pathway affiliation of the genes was identified using Ingenuity Systems Pathway Analysis software (Ingenuity Systems Inc.). The graph displays the top 10 identified pathways. The 542 genes were strongly associated with p53-mediated signaling (P = 0.000186). Orange squares indicate the ratio of the number of genes from the dataset that map to the canonical pathway. The solid blue bars correspond to the P-value representing the probability that the association between the genes and the identified pathway occurs by chance alone (calculated by Fisher's exact test with Benjamini-Hochberg multiple testing correction, given as a log P-value). The involvement of the p53 signaling pathway was statistically significant even at the level of individual genes. The mRNA levels of genes mediating (B) cellular DNA damage responses, (C) cell cycle arrest and (D) apoptosis were significantly altered in patients with HAM/TSP. Box plot represent median ± 1.5 IQR. P-values were calculated using a two-tailed Mann-Whitney test; Ctrl: n = 9, AC: n = 20, HAM/TSP: n = 10.

Gene expression profiles were generated from blood samples taken from patients with HAM/TSP, ACs and HTLV-1-seronegative individuals (Table S1 A). To identify a transcriptional signature that reflects HTLV-1 infection per se irrespective of clinical phenotype, we compared gene expression profiles between uninfected and all HTLV-1-positive individuals (Figure S1). HTLV-1 infection resulted in altered blood expression levels of 542 genes (Table S2). The majority of identified gene products were involved in cell cycle control, cell proliferation and anti-viral immune responses: the most strongly associated cellular process was the p53 signaling pathway (Figure 1 A). In accordance with previous *in vitro* findings [24], [25], mediators of cell cycle arrest and apoptosis were over-represented in patients with HAM/TSP while expression of molecules in the DNA damage response pathways was inhibited (Figure 1 B–D).

### The blood transcriptional signature of HAM/TSP pathology

**Figure 2:**
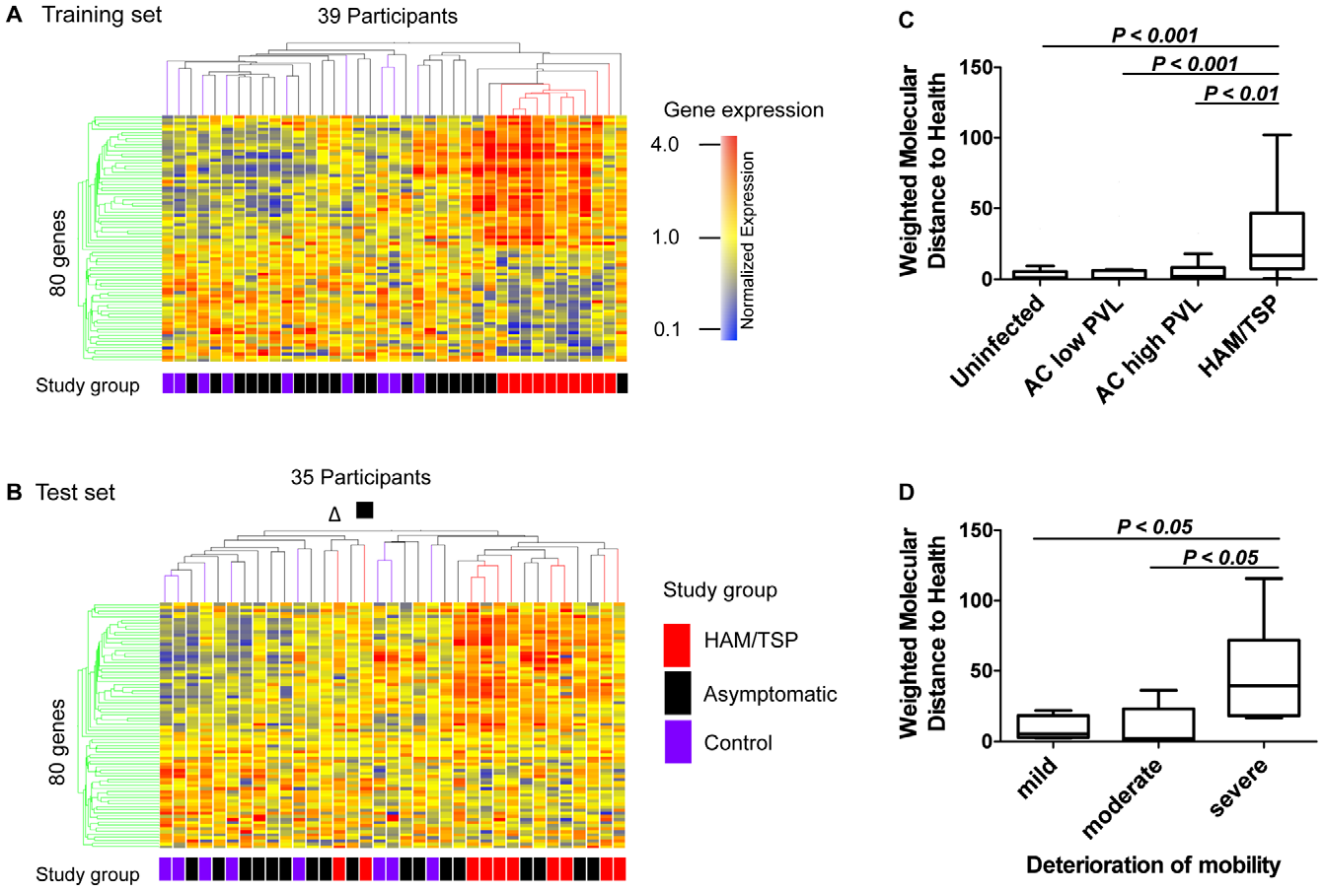
Presence of an 80-gene blood transcriptional signature in HAM/TSP. The blood expression levels of 80 genes differed between patients with HAM/TSP and clinically asymptomatic HTLV-1 carriers and uninfected individuals. (A) Microarray training and (B) test set gene expression profiles. Participants were clustered in hierarchical condition trees (Spearman correlation with average linkage) with each row representing a single gene and each column an individual subject. Color scale indicates normalized expression values and colored boxes below the trees indicate the individual's clinical classification (study group). ACs with high or low HTLV-1 proviral load were not distinguished by color because their results did not differ significantly either in hierarchical clustering or WMDH analysis. (C) Quantification of transcriptional changes in the 80 genes. The ‘weighted molecular distance to health’ metric [12] was calculated for each patient and the median compared between study groups (Mann-Whitney test). (D) Correlation between the transcriptional signature and the clinical severity of HAM/TSP. Extent of impaired mobility was assessed by patients' performance in the 10-meter walk test (see methods). Graphs (C) and (D) comprise data of the training and test set. PVL = HTLV-1 proviral load. Box plot represent median ± 1.5 IQR. The P-values were calculated using a two-tailed Mann-Whitney test.

Differential gene expression in HTLV-1 infection is driven both directly, by the viral infection of T cells (in proportion to the proviral load, i.e. the percentage of HTLV-1-infected PBMCs), and by the presence or absence of the inflammatory disease HAM/TSP. To identify genes associated with HAM/TSP whose expression varied independently of proviral load, we subdivided ACs into two groups: those with high proviral load (≥1% PBMCs) or low proviral load (<1% PBMCs). A distinct 80-gene blood transcriptional signature in patients with HAM/TSP was identified by non-parametric group comparison (Table S3). Hierarchical clustering analysis based on similarity in gene expression grouped individuals into two clusters associated with presence or absence of the inflammatory disease (two-tailed Fisher's exact test: P<0.0001) but not gender, age and ethnicity (Figure 2 A).

### Replication of the HAM/TSP transcriptional signature in a validation cohort

To validate the HAM/TSP transcriptional signature identified in the first cohort (training set), we applied the list of 80 genes to a second, independently collected and processed patient cohort (test set; Figure 2 B; Table S1 B). Based on the expression levels of these 80 genes, *k*-nearest neighbor class prediction identified patients with HAM/TSP in the test set with a sensitivity of 70% and specificity of 92%; a support vector machine approach gave similar results (Table S4).

Two ACs in the training set and three in the test set were misclassified as HAM/TSP, representing 10% and 17% of the respective asymptomatic cohorts. However, three of these five individuals had previously presented with urticaria and urinary urgency, which are associated with onset of HAM/TSP, and with episodes of uveitis, and other inflammatory conditions associated with HTLV-1 [26]. These observations also suggest that the transcriptional signature is specifically associated with HTLV-1-associated inflammatory disease, rather than viral infection alone.

Transcriptional changes in the 80-gene signature were quantified using the ‘weighted molecular distance to health’ (WMDH) statistic [12] which measures the difference in expression of specified genes between patients and controls. The WMDH was significantly higher in patients with HAM/TSP than in ACs with high or low proviral load, confirming the association between the transcriptional signature and the inflammatory disease (Figure 2 C).

### Intensity of the blood transcriptional signature correlates with clinical severity of HAM/TSP

Two patients diagnosed with HAM/TSP clustered with uninfected and asymptomatic individuals in the test set (Figure 2 B: filled triangle, empty square). To test whether this observation reflected heterogeneity in disease severity, we grouped patients according to their performance in the 10-meter timed walk, a test of the patient's mobility, on the day the blood sample was taken (Figure 2 D). Indeed, the two outlier patients had only mild or moderate impairment of mobility. In the whole cohort, the WMDH was significantly greater in patients who took >30 s to walk 10 m or required wheelchairs compared with less disabled HAM/TSP patients (healthy controls <8 s). Thus, the degree of molecular perturbation of the transcriptional signature reflected the clinical severity of HAM/TSP (Figure 2 D).

### The gene expression profiles of HAM/TSP and multiple sclerosis are distinct

**Figure 3:**
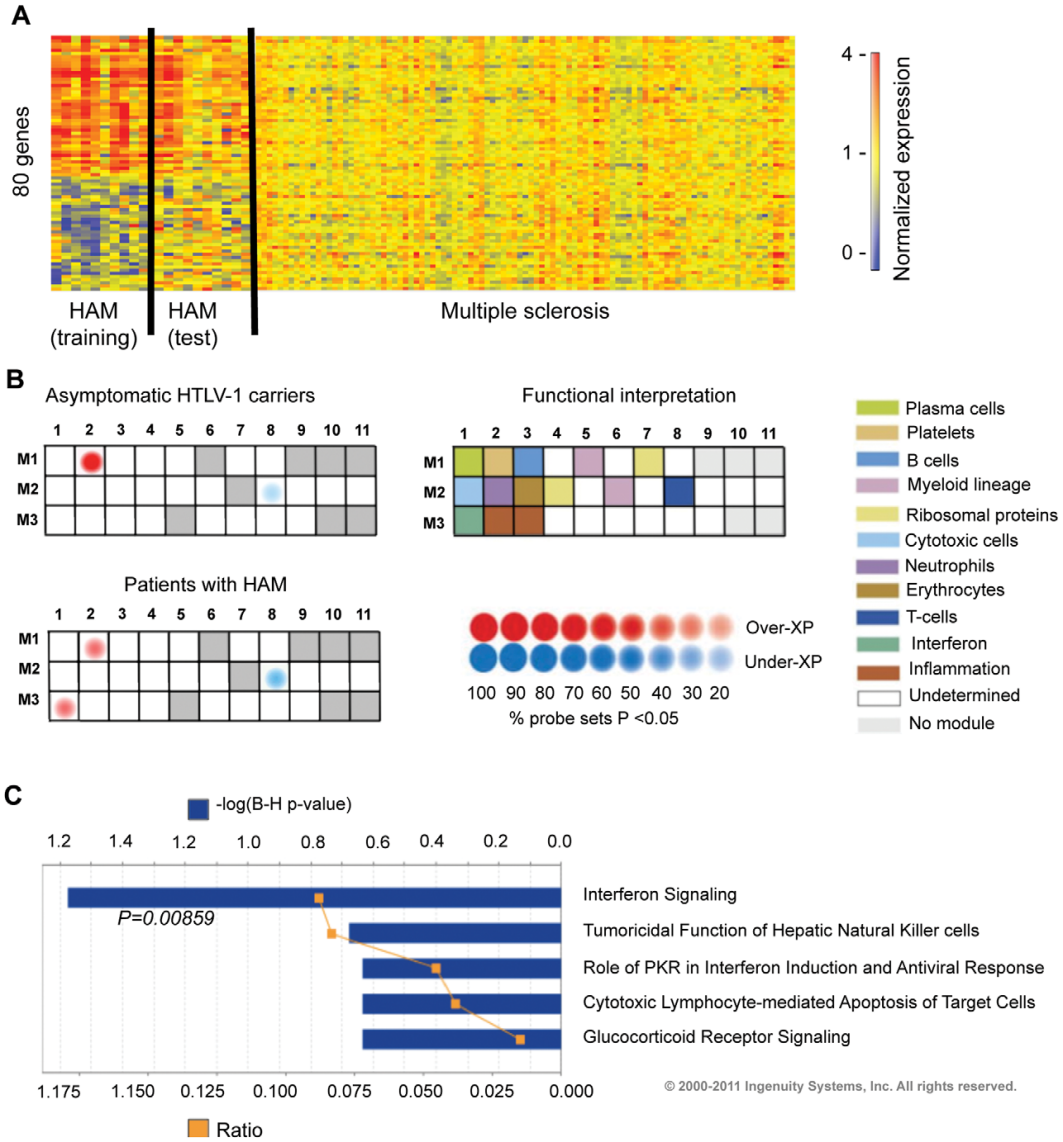
The HAM/TSP transcriptional signature comprises IFN-stimulated genes and is not present in multiple sclerosis. (A) The 80-gene transcriptional signature discovered in HAM/TSP was not present in patients with multiple sclerosis (MS). Datasets were normalized to their own controls (training set: HAM/TSP n = 10, Ctrl n = 9; test set: HAM/TSP n = 10, Ctrl n = 8; multiple sclerosis data set [27]: patients n = 99, Ctrl n = 45). (B) Modular analysis approach. Gene expression levels were compared between HTLV-1-positive study groups and healthy control subjects on a module-by-module basis [28]. Modules correspond to previously identified co-regulated gene clusters that were assigned biological functions by unbiased literature profiling (see legend grid on the right). Over-abundance of transcripts in a module is depicted in red, under-abundance in blue. The intensity of the dot corresponds to the percentage of genes in the respective module that are significantly differentially expressed between the study groups. (C) Canonical pathway analysis of the 80-gene HAM/TSP signature (Ingenuity Systems Pathway Analysis software). Orange squares represent the ratio of the number of genes present in the transcriptional signature versus all genes in the pathway. Solid blue bars correspond to the P-value representing the probability that the association between the genes and the identified pathway is due to chance alone (Fisher's exact test with Benjamini-Hochberg multiple testing correction, given as log P-value).

Clinically, HAM/TSP closely resembles certain forms of multiple sclerosis; both diseases show neurological abnormalities due to axonal lesions in the spinal cord and brain [1], [5]. We therefore compared the present data with the recently reported whole blood gene-expression profiles from 99 patients with multiple sclerosis [27]. Employing the *k*-nearest neighbor class prediction approach, using the 80-gene list, none of the multiple sclerosis patients were classified as having HAM/TSP. Thus, the signature distinguished HAM/TSP from the clinically similar condition multiple sclerosis (Figure 3 A).

### Over-expression of IFN-stimulated genes in patients with HAM/TSP

To explore the biological function of the observed transcriptional changes in HAM/TSP, we employed a modular analysis framework [28] that compares transcript abundance between patients and healthy control subjects in 28 groups (“modules”) of co-regulated genes, the majority of which have been shown to reflect specific cell populations or biological processes. The modular blood signature (Figure 3 B) revealed an over-expression of IFN-stimulated genes in HAM/TSP that was absent in asymptomatic virus carriers (module M3.1). Differentially expressed genes included key proteins of IFN signaling (*STAT1, STAT2*), genes associated with antigen processing (*TAP-1*), cell migration (*CXCL10*) and the innate antiviral response (*IFI35*). Transcriptional changes in the IFN-stimulated genes were reproduced in the test set and were independent of proviral load (Figure S2).

A separate pathway-focused analysis of the 80-gene blood transcriptional signature, using Ingenuity Systems Pathway Analysis software, independently confirmed IFN signaling as the dominant pathway associated with HAM/TSP (Figure 3 C). Here, IFN-stimulated transcripts constituted 29% (23 genes) of the 80 genes, and included genes inducible by both type I (IFN- α/β) and type II (IFN-γ) IFNs.

To test whether the expression of the distinct subset of IFN-stimulated genes in the 80-gene blood transcriptional signature reflects common immune responses observed in several diseases, we quantified expression of these genes in bacterial and other inflammatory diseases (Figure S3 A). No significant transcriptional changes in IFN-stimulated genes were detected in patients with multiple sclerosis, Still's disease or individuals infected with Staphylococcus or group A Streptococcus. Expression levels in adults or children with systemic lupus erythematosus (SLE) were similar to those observed in patients with HAM/TSP; however, both adult and pediatric SLE differentially expressed 92–95% of IFN-stimulated genes comprised in the IFN module (M3.1), whilst the IFN response in patients with HAM/TSP was limited to a small subset (Figure S3 B). We conclude that in contrast to the broad IFN-stimulated gene expression pattern in SLE, the IFN-mediated immune response in patients with HAM/TSP is restricted to a distinct subset of IFN-stimulated genes.

To confirm that the observed HAM/TSP transcriptional signature resulted from differential transcriptional regulation rather than differences in cell composition, we quantified frequencies of peripheral leukocyte subsets in HTLV-1 carriers and control subjects by flow cytometry. No significant differences in the abundance of antigen-presenting or major effector cell populations were observed between the study groups (Figure S4).

### The HAM/TSP IFN-inducible signature is present in monocytes and neutrophils

**Figure 4:**
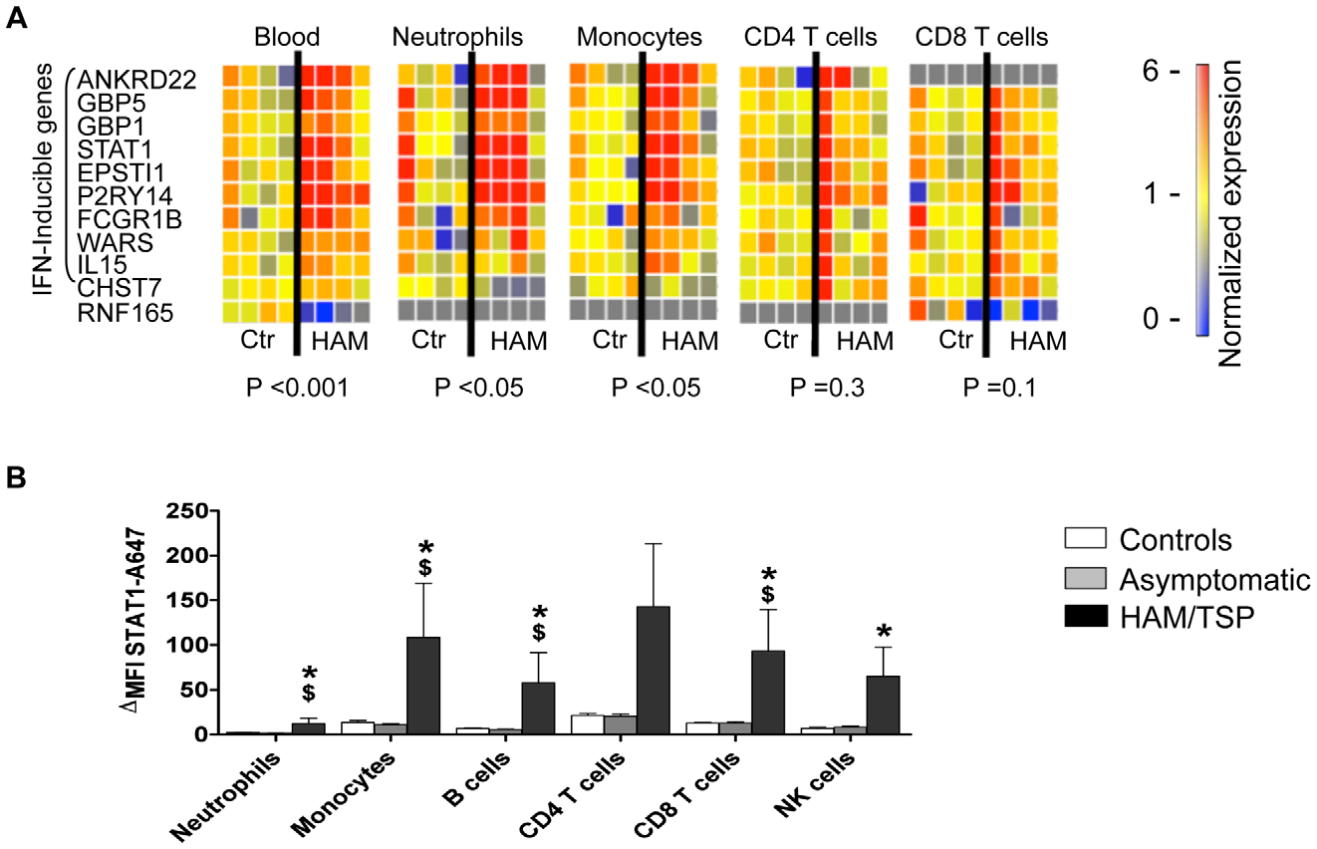
Over-expression of IFN-stimulated genes in patients with HAM/TSP. (A) The mRNA levels of the 11 genes most strongly associated with presence of HAM/TSP in whole blood and separated peripheral blood immune cell populations as quantified by real-time PCR (P-value: Fisher's method of combining probabilities). Ctrl: n = 4, HAM/TSP: n = 4. (B) STAT1 protein levels in blood leukocyte populations. Whole blood samples were stained for markers of leukocyte populations; intracellular expression of STAT1 was assessed by flow cytometry. P-values were calculated using a two-tailed Mann-Whitney test with P<0.05 HAM/TSP versus ^*^controls or ^$^AC. Data represent mean ± SEM, Ctrl: n = 6, AC: n = 6, HAM/TSP: n = 6.

CD4^+^ T cells constitute the main reservoir for HTLV-1 *in vivo*. To test whether the observed IFN-inducible signature was restricted to HTLV-1-infected CD4^+^ T cells we selected genes with the highest association with HAM/TSP (11 genes; see Methods) and quantified their expression levels in separated blood leukocyte populations by quantitative RT-PCR (Figure 4 A). The IFN-inducible signature was present in neutrophils and monocytes (Fisher's method of combining probabilities: P = 0.0271 and P = 0.0260 respectively) but not in CD4^+^ or CD8^+^ T cells (P = 0.3 and P = 0.1, respectively, Figure 4 A). The mRNA levels of four representative IFN-stimulated genes (STAT1, CD64, FAS and CXCL10, Figure S5) correlated well with their protein levels, thus confirming the presence of the IFN-inducible signature at the protein level. Furthermore, increased protein levels of STAT1, a key molecule in IFN signaling, were detected by flow cytometry in all peripheral blood effector cell populations from patients with HAM/TSP, but not in ACs or uninfected controls (Figure 4 B). These results suggest that the observed IFN-inducible transcriptional signature in whole blood did not result from direct activation of IFN-mediated anti-viral pathways in infected CD4^+^ T cells but rather that all cell types had been systemically exposed to IFNs *in vivo*.

### Molecular basis of the IFN-inducible signature in HAM/TSP

To identify the molecular basis of the IFN-inducible signature, we tested for abnormal production of IFNs and abnormal responses to IFNs in patients with HAM/TSP. In agreement with previous findings [29], [30] we found that protein levels of the IFN-γ-inducible chemokine CXCL10 were significantly increased in plasma from patients with HAM/TSP compared to ACs; however, IFN-α2, IFN-β, IFN-λ and IFN-γ protein levels were not elevated (Figure S6). There were no differences in the production of IFN-α by stimulated plasmacytoid dendritic cells in short-term whole blood *ex vivo* assays. The frequency of CD8^+^ T cells producing IFN-γ in patients with HAM/TSP was greater than that in uninfected controls, but did not differ significantly from the frequency observed in ACs (Figure S7).

**Figure 5:**
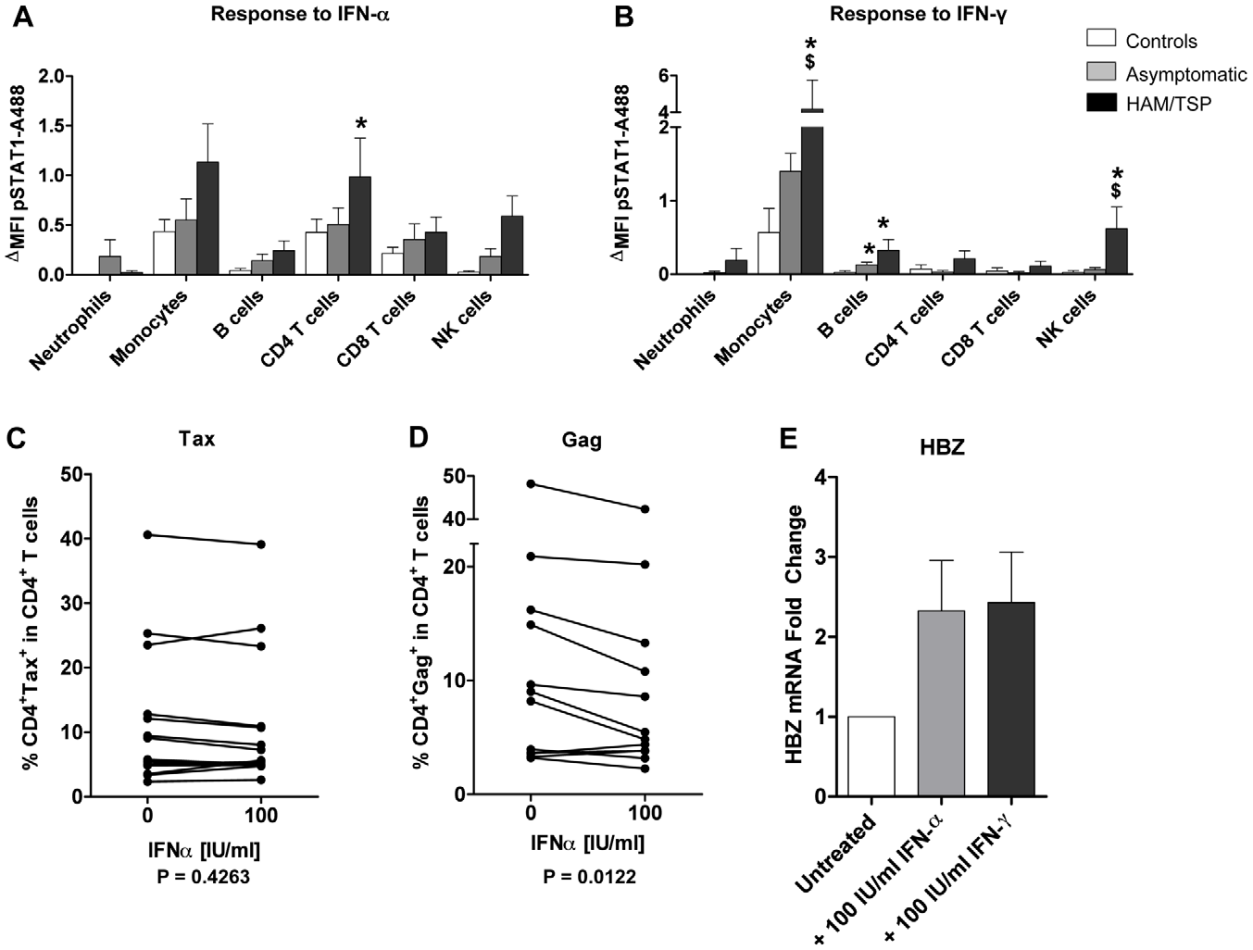
Cellular and viral responses to IFN in HTLV-1 carriers. STAT1-P(Y701) phosphorylation in response to (A) IFN-α and (B) IFN-γ was quantified in whole blood by flow cytometry. Bar graphs represent the mean ± SEM. P-values were calculated using a two-tailed Mann-Whitney test with *P<0.05 versus controls, ^$^P<0.05 versus AC; Ctrl: n = 6, AC: n = 6, HAM/TSP: n = 6. Effect of exogenous IFN-α on the expression of viral transcriptional transactivator (C) Tax (P = 0.4263), (D) the structural protein Gag (P = 0.0112) and (E) HBZ mRNA (P = 0.0742). Tax and Gag protein levels were assessed by flow cytometry, HBZ mRNA was quantified by real-time PCR. P-values were calculated using a paired Wilcoxon signed-rank test; Tax and Gag protein: HAM/TSP: n = 11; HBZ mRNA: AC: n = 2, HAM/TSP: n = 7.

Next we analyzed molecules of the IFN signaling pathway that are involved in IFN-responsiveness. Surface expression levels of IFN-α and IFN-γ receptors on peripheral leukocyte populations were similar in all study groups (Figure S8). We then measured phosphorylated STAT1 (p-STAT1) by flow cytometry as a marker of activation of the IFN signaling pathway at the single-cell level. Stimulation with recombinant IFNs (particularly IFN-γ) led to an increase in p-STAT1 levels in all study groups (Figure 5 A–B). Patients with HAM/TSP had higher STAT1 protein baseline levels which after IFN stimulation resulted in significantly higher p-STAT1 levels compared to ACs or uninfected controls. Expression of STAT1 itself is upregulated by IFNs [31]; it is likely that exposure to IFNs *in vivo* induces higher STAT1 protein and thus p-STAT1 levels which makes leukocytes from patients with HAM/TSP more sensitive to IFNs.

### Effect of IFN-α and IFN-γ on HTLV-1 Tax, Gag and HBZ expression

IFNs activate several intracellular antiviral responses and are typically associated with a beneficial effect on the outcome of viral infection [32]. To examine the effect of IFNs on HTLV-1 protein expression, we quantified expression of HTLV-1 Tax and Gag after treatment with exogenous IFN-α and IFN-γ. Interestingly, whilst IFN-α treatment partially inhibited the expression of the HTLV-1 protein Gag, it could not suppress production of the pleiotropic viral transcriptional transactivator Tax (Figure 5 C–D). In contrast, IFN-γ treatment did not alter the expression of either Gag or Tax proteins in infected CD4^+^ T cells (Figure S9 A, B). Exogenous IFN-α and IFN-γ increased HBZ mRNA levels but this did not reach statistical significance (Figure 5 E; Figure S9 C, D).

## Discussion

Despite the growing understanding of the mechanisms of HTLV-1 persistence, little is known about the pathogenesis of HAM/TSP and importantly, what distinguishes hosts who develop the disease from those who remain asymptomatic. The aim of this study was to identify biological pathways and molecules in whole blood gene expression profiles of asymptomatic HTLV-1 carriers and patients with HAM/TSP to generate new hypotheses on the mechanisms of viral persistence and the pathogenesis of HAM/TSP pathology.

We found that the presence of HAM/TSP was associated with over-expression of a distinct subset of IFN-stimulated genes in circulating leukocytes. The IFN-inducible signature was absent in healthy HTLV-1 carriers and correlated positively with the clinical severity of the inflammatory disease. Gene expression signatures may reflect either differences in the frequencies of specific cell populations or a difference in gene expression at the single-cell level. We observed no significant differences in the frequencies of the blood cell populations (Figure S4): we conclude that the observed IFN-inducible signature resulted from a difference in the intensity of expression of IFN-stimulated genes at the single-cell level.

Expression of IFN-stimulated genes was evident in neutrophils and monocytes, but not in T cells, which are the cellular reservoir of HTLV-1 *in vivo* [15]. It is therefore likely that the HAM/TSP-specific transcriptional profile originated from exposure to IFNs *in vivo* rather than direct activation of the IFN-response pathway in infected cells. The IFN-stimulated genes identified in HAM/TSP can be induced by both type I and type II IFNs [33] and we did not detect abnormal plasma levels of type I or type II IFNs in patients with HAM/TSP, perhaps because of their short half-lives in blood [34]. However, CD8^+^ T cells from patients with HAM/TSP produced high levels of IFN-γ in response to a non-specific stimulus *ex vivo*, which may reflect higher local production of IFN-γ *in vivo*.

IFN-stimulated genes have been shown to mediate potent antiviral and neuroprotective effects [32], [35]. However, several recent studies have implicated IFNs in the pathogenesis of autoimmune disorders such as rheumatoid arthritis [36], systemic lupus erythematosus [37], systemic sclerosis [38] and Sjögren's syndrome [39]. It has been hypothesized that IFNs trigger autoimmunity by promoting the activation and survival of autoreactive T and B cells in genetically predisposed individuals [40]. However, there is little evidence for antigen-specific autoreactivity in HAM/TSP [41]; instead, the risk of disease is linked to high frequencies of poor quality antiviral cytotoxic T cells [42]; i.e. low avidity of HTLV-1 antigen recognition and reduced lytic efficiency. IFNs may contribute to immunopathological processes in the central nervous system by stimulating and propagating a detrimental immune response to HTLV-1; however, the precise pathways responsible have yet to be determined.

Despite similarities in clinical presentation the gene expression profiles of HAM/TSP and multiple sclerosis were distinct. The IFN-inducible signature present in HAM/TSP was absent in multiple sclerosis, suggesting that different pathogenetic mechanisms contribute to the axonal damage observed in both diseases. A recent study reported the expression of a subset of type I IFN-stimulated genes in 50% of patients with a particular clinical subtype of multiple sclerosis (relapsing-remitting) [43]. However, in agreement with our findings there was little overlap between the IFN-stimulated genes identified in HAM/TSP and the genes whose expression was altered in multiple sclerosis. Furthermore, we found that different subsets of IFN-stimulated genes are actively expressed in other diseases with IFN-inducible signatures (Figure S3). There are more than 300 known IFN-stimulated genes, the individual actions of many of which are largely uncharacterized. Thus, it is conceivable that there are cell-type-, pathogen- and disease-specific patterns of IFN-stimulated gene expression that contribute to a beneficial or detrimental outcome of immunological processes.

Type I IFN therapy is effective in controlling certain persistent viral infections [44], and there is evidence that it can slow down disease progression in multiple sclerosis [45]. However, clinical trials assessing the benefit of IFN-α and IFN-β therapy in the treatment of HAM/TSP have been less successful [46]; few patients showed any improvement and the overall clinical benefit was limited. Although a beneficial net effect of systemically administered IFN cannot be excluded, two findings in the present study support the clinical observation that IFNs do not effectively control HTLV-1 infection: (i) over-expression of IFN-stimulated genes was not observed in healthy HTLV-1 carriers who efficiently control the infection; (ii) while IFN-α reduced the expression of the structural viral protein Gag as reported previously [47], we found that IFN treatment did not affect the expression of the viral transactivator protein Tax or HBZ mRNA. HTLV-1 Tax is essential to the viral life cycle as it has been shown to drive viral replication, promote host cell proliferation and viral cell-to-cell spread [48], [49]. HBZ has been shown to down-modulate Tax function [50], [51] whilst promoting infectivity and viral persistence [52]. A recent study has linked elevated HBZ mRNA levels to increased clinical severity of HAM/TSP [53]. We found that IFN treatment increased HBZ expression but this observation did not reach statistical significance. We conclude that although type I IFN treatment may decrease the production of viral structural complexes, i.e. capsids, it does not completely abrogate viral activity and proliferation of infected cells. In addition, HTLV-1 viral proteins have been reported to manipulate and evade the IFN response by suppressing interferon regulatory factors and up-regulating suppressors of IFN signaling [16], [54], [55].

Perturbations of the p53 signaling pathway were evident in all HTLV-1 infected individuals, irrespective of disease status. The p53 signaling pathway prevents cell proliferation of genomically unstable cells by promoting cell cycle arrest, senescence and apoptosis [56]. Altered p53 signaling could reflect a highly active immune response with rapid cell turnover and clonal expansion. However, abnormal p53 signaling has been implicated in HTLV-1-mediated leukemogenesis [49]. The p53 protein itself is usually intact in HTLV-1 leukemic cells, but there is evidence that the virus inhibits p53-induced anti-viral responses by targeting other components of the p53 signaling pathway [57], [58]. This hypothesis is supported by our finding that HTLV-1-positivity was associated with low mRNA levels of molecules involved in the detection of DNA damage, i.e. the ataxia telangiectasia mutated protein (ATM) and the DNA-dependent protein kinase (PRKDC). The protein products of these genes sense dsDNA breaks due to viral integration into the cell's genome and induce cell cycle arrest and/or apoptosis [59]. Decreased expression of ATM and PRKDC could therefore facilitate the survival of genomically unstable HTLV-1-infected cells and allow premature DNA replication in the presence of genomic lesions, thus contributing to the oncogenic potential of HTLV-1.

In summary, our study revealed that over-expression of a specific subset of IFN-stimulated genes distinguishes patients with HAM/TSP from asymptomatic HTLV-1 carriers, which indicates an unexpected role of IFN-stimulated genes in the pathogenesis of central nervous system inflammation in HTLV-1 infection. This finding opens new avenues for research on HAM/TSP pathogenesis and new areas of therapeutic intervention.

## Materials and Methods

### Ethics statement

All study participants have given written informed consent. Ethical approval for this study was obtained as follows: IRB approval numbers: St Mary's NHS Trust Local Research Committee EC no. 02.31 (27.06.2002); National Research Ethics Service, Oxfordshire REC C no. 09/H0606/106 (16.11.2009).

### Study participants

Consenting participants attended the HTLV clinic at St Mary's Hospital, London, UK. Deterioration of mobility in patients with HAM/TSP was assessed by a 10-meter timed walk (time taken to walk 10 m with or without a walking aid). The extent of deterioration was categorized as ‘mild’ (8–15 s; n = 5), ‘moderate’ (16–30 s; n = 6) or ‘severe’ (>30 s or use of a wheelchair; n = 6).

### HTLV-1 proviral load measurement

HTLV-1 proviral load was measured as described previously [6] and expressed as percentage of peripheral blood mononuclear cells (PBMCs).

### Sample processing and microarray analysis

Blood was collected in Tempus tubes (Applied Biosystems, Foster City, CA, USA). Total RNA and cDNA were prepared as described previously [12] and analyzed on HumanHT-12 V3 or WG6 V3 expression BeadChip arrays (Illumina, San Diego, CA, USA). Raw gene expression values were normalized per gene to the median gene expression value across all samples and supervised non-parametric analysis was carried out in GeneSpring GX7.3.1 (Agilent Technologies, Edinburgh, UK). A series of filters was applied to identify genes differentially expressed between ACs and patients with HAM/TSP (Figure S2). To identify the genes most strongly associated with HAM/TSP, we selected genes that were >1.5-fold differentially expressed versus ACs in >50% of patients with HAM/TSP (11 genes). To visualize transcriptional patterns, an iterative agglomerative clustering method was applied to gene lists (algorithm: Pearson correlation) and samples (algorithm: Spearman correlation). Microarray data was deposited in the Gene expression Omnibus database (accession number GSE29312).

Illumina HT12 V3 microarray data relating to other diseases has been published previously and is publically available: multiple sclerosis [27], Still's disease [12], Staphylococcus or group A Streptococcus infection [12], adult and pediatric systemic lupus erythematosus [12].

Class prediction was performed within GeneSpring using the *k*-nearest neighbor algorithm (neighbors = 10; P-value ratio cut off = 0.65) or support vector machine (Gaussian radial basis, diagonal scaling = 3).

### Biological interpretation of transcriptional signatures

Canonical pathway analysis was performed using the web-based Ingenuity Systems Pathway Analysis software (Ingenuity Systems Inc., Redwood City, CA, USA). Modular transcriptional fingerprints [28] for the HTLV-1-positive study groups were generated comparing BeadStudio raw gene expression values to baseline median expression values of uninfected controls for all genes present in a module (Student t-test, P<0.05). The weighted molecular distance to health (WMDH) was calculated as described previously [12] to quantify global transcriptional changes relative to a pre-determined baseline value.

IFN-stimulated transcripts in the 80-gene blood transcriptional signature were identified using the Interferome database (www.interferome.org) [33].

### Isolation of separated cell populations, RNA extraction and quantitative Real-Time PCR

Neutrophils (CD15^+^) followed by CD8^+^ T cells or monocytes (CD14^+^) followed by CD4^+^ T cells were isolated from whole blood using antibody-coupled magnetic whole blood beads (Miltenyi Biotec, Bergisch Gladbach, Germany). RNA from blood and isolated cell populations was purified using the QIAamp RNA Blood Mini Kit (QIAGEN, Hilden, Germany).

Total cDNA was generated from 1 µg of RNA using the SuperScript III First-Strand Synthesis Supermix (Invitrogen, Paisley, UK). Custom Taqman Low Density Array cards (Applied Biosystems, Carlsbad, CA, USA) comprised probes for the top 11 genes associated with HAM/TSP (Table S5) and six reference genes for normalization (*B2M, ACTB, HMBS, GNB2L1, RPLP0, 18S*). qRT-PCR was performed on a 7900HT fast real-time system (Applied Biosystems). Ct values were extracted and analyzed using SDS2.3 software (Applied Biosystems).

### Quantification of IFN concentrations in plasma

Plasma concentrations of IFNs were measured by Luminex using a LEGENDplex assay kit according to the manufacturer's instructions (Bioplex, Biolegend, San Diego, USA). Bioactivity of type I and III IFNs in plasma samples was assessed using the iLite Human Interferon Alpha Kit (Neutekbio, Galway, Ireland).

### Quantification of blood leukocyte populations and CD64 and FAS expression by flow cytometry

Fresh blood was stained with monoclonal antibodies for population specific markers (neutrophils: CD45^+^CD14^−^CD16^+^, monocytes: CD45^+^CD14^+^CD16^−^, B cells: CD45^+^CD19^+^CD20^+^, T cells: CD45^+^CD3^+^CD4^+^ or CD8^+^, NK cells: CD45^+^CD3^−^CD56^+^CD16^+^) and CD64 or FAS antibodies for 15 min at room temperature (RT). Following lysis of erythrocytes with BD FACS Lysing solution (BD Biosciences, Franklin Lakes, NJ, USA), samples were acquired on a CyAn ADP flow cytometer (Beckman Coulter, Marseille, France) and analyzed using FlowJo software (TreeStar, Ashland, USA). All antibodies were purchased from eBioscience, Beckman Coulter, BD Pharmingen and Miltenyi Biotec.

### Detection of IFN-α secretion and IFN-γ production in whole blood by flow cytometry

IFN-α secretion assay: blood samples were stimulated with 10 µM R-848 (TLR-7/8 agonist; Insight Biotech, Wembley, UK) for 3 h at 37°C. IFN-α secreted by plasmacytoid dendritic cells (BDCA-2^+^CD123^+^) was measured using the Miltenyi Human IFN-α Secretion Assay Detection Kit. Intracellular IFN-γ production: blood samples were stimulated with 10 ng/ml PMA, 0.5 µg/ml calcimycin (Sigma, Gillingham, UK) in the presence of monensin (eBioscience, San Diego, CA, USA) for 4 h at 37°C and analyzed by flow cytometry.

### Quantification of IFN receptor surface expression in whole blood by flow cytometry

Fresh whole blood samples were incubated with IFNGR2-Fluorescein (R&D Systems, Minneapolis, MN, USA), IFNGR1-PE (eBioscience) or IFNAR1-Fluorescein and IFNAR2-PE (PBL Interferon Source, Piscataway, NJ, USA) and immune cell population markers for 25 min at RT. Following lysis of erythrocytes with BD FACS Lysing solution (BD Biosciences), samples were acquired on a CyAn ADP flow cytometer (Beckman Coulter) and analyzed using FlowJo software (TreeStar).

### Detection of basal STAT1 protein levels and IFN-induced STAT1 phosphorylation by flow cytometry

Heparinized whole blood was treated with 1000 IU/ml of recombinant IFN-α2a or IFN-γ (PBL Interferon Source) for 20 min at 37°C. Detection of STAT1 phosphorylation by flow cytometry was adapted for whole blood following the specifications refined by Chow and colleagues [60].

### Effect of exogenous IFN on Tax and Gag protein and HBZ mRNA expression

CD8^+^ T cells were depleted from PBMCs by positive selection using antibody-coated magnetic microbeads (Miltenyi Biotec) to prevent cytotoxic T cell-mediated killing of HTLV-1 expressing cells. Cells were incubated in the presence of 100 IU/ML IFN-α, 100 IU/ml IFN-γ or PBS in RPMI 1640 medium (Sigma) supplemented with 10% FCS, 2 mM L-Glutamine, 100 U/ml Penicillin and 100 µg/ml Streptomycin (Life Technologies) at 37°C in 5% CO_2._ For HBZ mRNA expression cells were harvested after 0, 2, 4, 8, and 16 h and RNA extracted using the RNeasy kit (QIAGEN, Hilden, Germany) according to the manufacturer's instructions. The cDNA was produced using the QuantiTect Reverse Transcription kit (QIAGEN) and qRT-PCR was performed in 96-well plates on a 7900HT fast real-time system (Applied Biosystems). Primer sequences detected spliced HBZ mRNA: forward primer 5′-GGACGCAGTTCAGGAGGCAC-3′, reverse primer 5′-CCTCCAAGGATAATAGCCCG-3′. Ct values were extracted and analyzed using SDS2.3 software (Applied Biosystems). HBZ mRNA levels were normalised to endogenous GAPDH levels (primers: forward 5′-CTTTGGTATCGTGGAAGGACTC-3′, reverse 5′-GTAGAGGCAGGGATGATGTTCT-3′.

For Tax and Gag protein detection cells were fixed and permeabilized after 16 h using the FoxP3 staining buffer set from eBioscience and stained for CD3, CD4, CD8, HTLV-1 Tax (LT4 antibody) [61] and HTLV-1 Gag (Gin7 antibody) [62] and analyzed by flow cytometry.

### Statistical analysis

Correlations were tested by Spearman's rank-order and differences between two study groups by Mann-Whitney *U* test. Pairwise comparisons were tested using the Wilcoxon signed-rank test. All tests were two-tailed. Box plots are depicted as median with error bars within the 1.5 interquartile range (IQR); bar graphs depict median with error bars representing the standard error of the mean (SEM). Statistical programs used included SPSS version 17; Microsoft Excel 2007; GeneSpring GX7.3.1 and the DiagnosisMed package in R.

## Supporting Information

Figure S1 **Data dimension reduction by supervised non-parametric analysis of the microarray training set.**
(TIF)

Figure S2 **Modular framework analysis of the HTLV-1 test set.** Gene expression levels were compared between (A) ACs or (B) patients with HAM/TSP and healthy control subjects on a module-by-module basis (Student t-test, P<0.05). Over-expressed genes are depicted in red, under-expressed genes in blue. The intensity of the dots corresponds to the percentage of genes that are significantly differentially expressed between the study groups. A functional interpretation of the modules is provided in the legend.
(TIF)

Figure S3 **Expression of a distinct subset of IFN-stimulated genes distinguishes HAM/TSP from other diseases.** (A) Transcriptional changes for all IFN-stimulated genes comprised in the 80-gene blood transcriptional signature were calculated using the WMDH metric and compared between patients with HAM/TSP (patients n = 20, control n = 17), multiple sclerosis (MS; n = 99, control n = 45), Still's disease (Still's; n = 31, control n = 22), individuals infected with Staphylococcus (n = 40, control n = 12) or group A Streptococcus (n = 23, control n = 12) and patients with adult (ASLE; n = 28, control = 15) or pediatric systemic lupus erythematosus (PSLE; n = 82, control = 18). P-values were calculated using a two-tailed Mann-Whitney test; box plots represent median ± 1.5 IQR. (B) In contrast to patients with SLE, transcriptional changes in patients with HAM/TSP were limited to a small subset of IFN-stimulated genes. Based on list of 76 IFN-stimulated genes in module M3.1, non-parametric group comparisons were performed between patients with HAM/TSP and adult or paediatric systemic lupus erythematosus (ASLE and PSLE). Numbers in brackets depict the number of genes that were significantly different expressed between patients with the disease and their respective healthy controls (two-tailed Mann Whitney test, P<0.05).
(TIF)

Figure S4 **Relative frequencies of peripheral blood cell populations in HTLV-1-positive patients and healthy controls.** Cells from heparinised blood were stained with monoclonal antibodies corresponding to well-characterised markers of antigen-presenting and effector cell populations. Following lysis of red blood cells using the BD FACS Lysing solution, the samples were analysed by flow cytometry. (A) plasmacytoid dendritic cells, (B) myeloid dendritic cells, (C) monocytes, (D) neutrophils, (E) B cells, (F) NK cells, (G) CD4^+^ T cells, (H) CD8^+^ T cells, (I) CD4^−^CD8^−^ T cells; WBC = white blood cells. P-values were calculated using a two-tailed Mann-Whitney test; Ctrl: n≤6, AC: n≤9, HAM/TSP: n≤9.
(TIF)

Figure S5 **Validation of the IFN-inducible transcriptional signature in HAM/TSP on the protein level.** The mRNA and protein levels of the IFN-stimulated genes (A, B) STAT1, (C, D) CD64 and (E, F) FAS were quantified by real-time PCR and flow cytometry on peripheral leukocyte populations. Data represents mean ± SEM; P-values were calculated using a two-tailed Mann-Whitney test with*P<0.05; mRNA: Ctrl: n = 4, HAM/TSP: n = 4; protein: Ctrl: n = 10, HAM/TSP: n = 10. (G) Blood mRNA levels of CXCL10 correlated well with CXCL10 protein levels in plasma (Spearman correlation P-value = 0.0011). AC: n = 13, HAM/TSP: n = 13.
(TIF)

Figure S6 **Plasma concentrations of Type I, II and Type III IFNs and CXCL10.** Plasma was cleared of blood cells by spin centrifugation and analyzed by luminex for concentrations of (A) IFN- α, (B) IFN-β, (C) IFN-γ, (D) IFN-λ and the IFN-γ-inducible chemokine (E) CXCL10. P-values were calculated using a two-tailed Mann-Whitney test. No Type I and Type III IFN bioactivity was detected in plasma samples (data not shown). Ctrl: n = 12, AC: n = 13, HAM/TSP: n = 13.
(TIF)

Figure S7 **Production of IFN-α and IFN-γ in HTLV-1 carriers. (**A) IFN-α secretion by plasmacytoid dendritic cells (pDCs) in whole blood in response to stimulation of TLR7 and TLR8. Fresh blood was stimulated with 10 µM R-848 for 3 h at 37°C. Secreted IFN-α was captured on the surface of pDCs and quantified by flow cytometry. (B) Intracellular production of IFN-γ in CD8^+^ T cells after PMA/calcimycin stimulation. Fresh blood was stimulated with 10 ng/ml PMA and 0.5 µg/ml calcimycin in the presence of monensin for 4 h at 37°C. Intracellular production of IFN-γ in CD8^+^ T cells was analyzed by flow cytometry. Histograms are gated on (A) pDCs or (B) CD8^+^ T cells; unstimulated controls are indicated as grey shaded area, stimulated samples are indicated by a solid black line. Data represents mean ± SEM; P-values were calculated using a two-tailed Mann-Whitney test; Ctrl: n = 6, AC: n = 6, HAM/TSP: n = 6.
(TIF)

Figure S8 **Surface expression of IFN receptors.** Heparinised blood samples were analysed by flow cytometry after staining with monoclonal antibodies corresponding to leukocyte populations and (A) IFN-α receptor 1 (IFNAR1), (B) IFN-α receptor 2 (IFNAR2), (C) IFN-γ receptor 1 (IFNGR1) or (D) IFN-γ receptor 2 (IFNGR2). No significant changes in IFN receptor abundance were detected between the study groups. Data represents mean ± SEM; P-values were calculated using a two-tailed Mann-Whitney test; Ctrl: n = 6, AC: n = 5, HAM/TSP: n = 4.
(TIF)

Figure S9 **Effect of exogenous IFN on Tax and Gag protein and HBZ mRNA expression.** Fresh PBMCs were depleted of CD8^+^ T cells and incubated for 16 h in the absence or presence of 100 IU/ml IFN-γ (Tax, Gag, HBZ) or IFN-α (HBZ). (A) Tax (P = 0.4263) and (B) Gag (P = 0.5561) protein levels were not altered by IFN-γ treatment as measured by flow cytometry. (C) HBZ mRNA levels were quantified by real-time PCR after 0, 2, 4, 8 and 16 h in response to IFN-α and IFN-γ treatment. Graph depicts mean ± SEM; AC: n = 2, HAM/TSP: n = 2. (D) Table depicts the average HBZ mRNA fold change after 16 h incubation with IFNs. P-values were calculated using a Wilcoxon signed-rank test. AC: n = 2, HAM/TSP: n = 7.
(TIF)

Table S1 **Demographics characteristics of uninfected and HTLV-1-positive individuals in the (A) microarray training and (B) test set.**
(DOC)

Table S2 **List of the 542 transcripts deregulated in HTLV-1 infection.**
(DOC)

Table S3 **List of 80 transcripts deregulated in HAM/TSP.**
(DOC)

Table S4 **Class prediction analysis of the 80-gene transcriptional signature.**
(DOC)

Table S5 **List of probes used in custom Taqman low density array.**
(DOC)
