# Supplementary figures and images for: Systems Biology Approaches Reveal a Specific Interferon-Inducible Signature in HTLV-1 Associated Myelopathy
Source: PLoS Pathog. 2012 Jan 26;8(1):e1002480. doi: 10.1371/journal.ppat.1002480 (PMC3266939; doi:10.1371/journal.ppat.1002480)

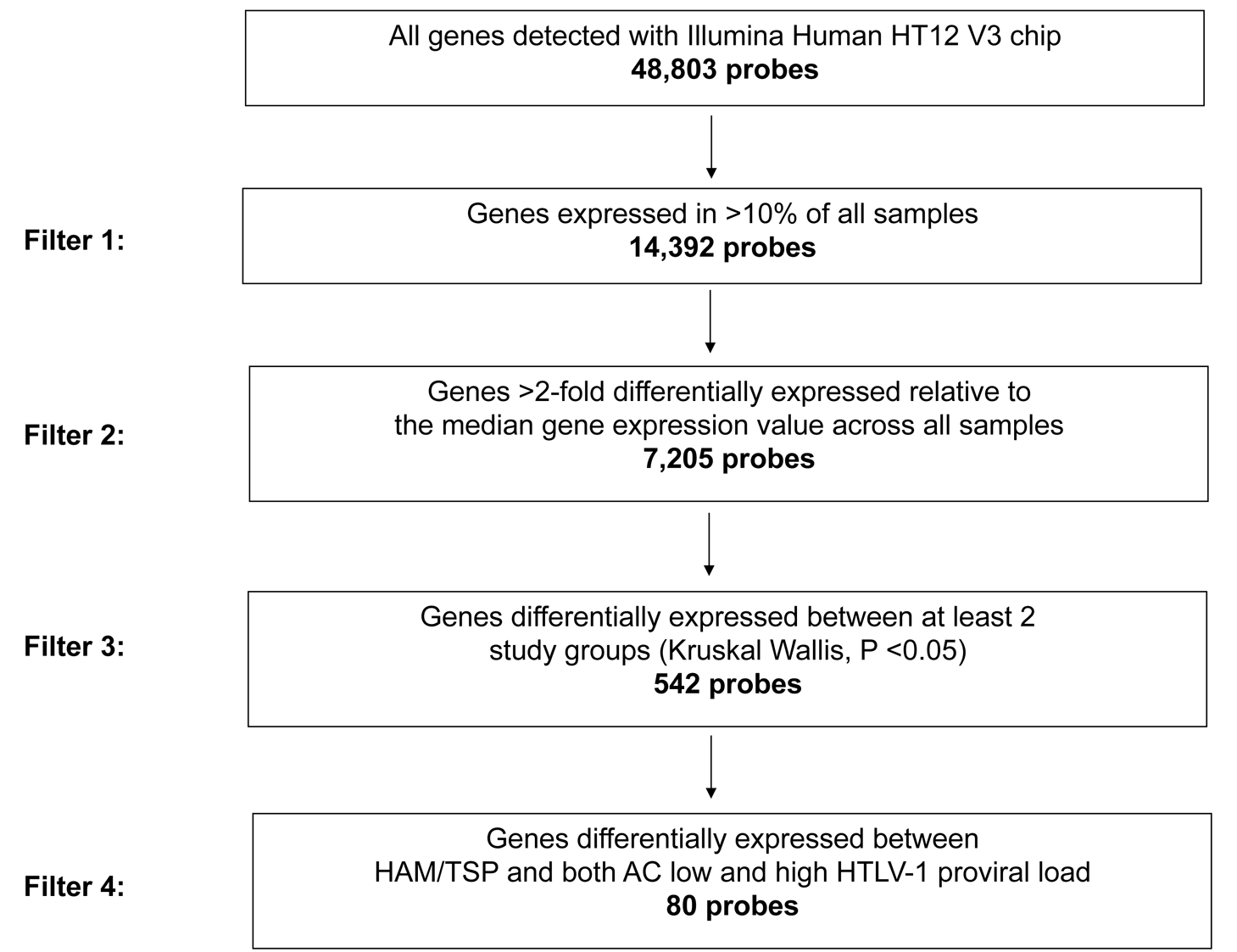

Supplement: Figure S1 — Data dimension reduction by supervised non-parametric analysis of the microarray training set. (TIF) [file ppat.1002480.s001.tif]

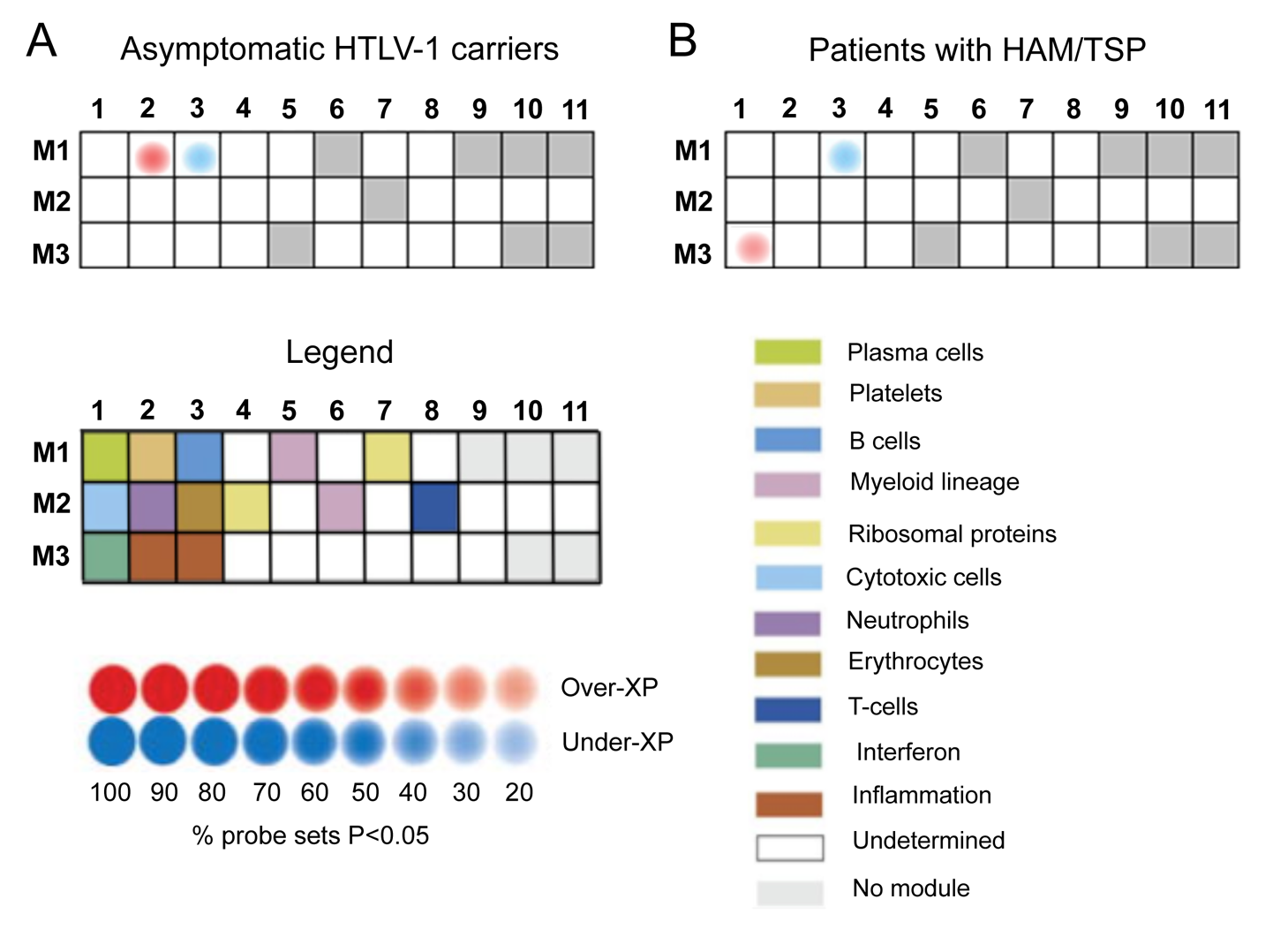

Supplement: Figure S2 — Modular framework analysis of the HTLV-1 test set. Gene expression levels were compared between (A) ACs or (B) patients with HAM/TSP and healthy control subjects on a module-by-module basis (Student t-test, P<0.05). Over-expressed genes are depicted in red, under-expressed genes in blue. The intensity of the dots corresponds to the percentage of genes that are significantly differentially expressed between the study groups. A functional interpretation of the modules is provided in the legend. (TIF) [file ppat.1002480.s002.tif]

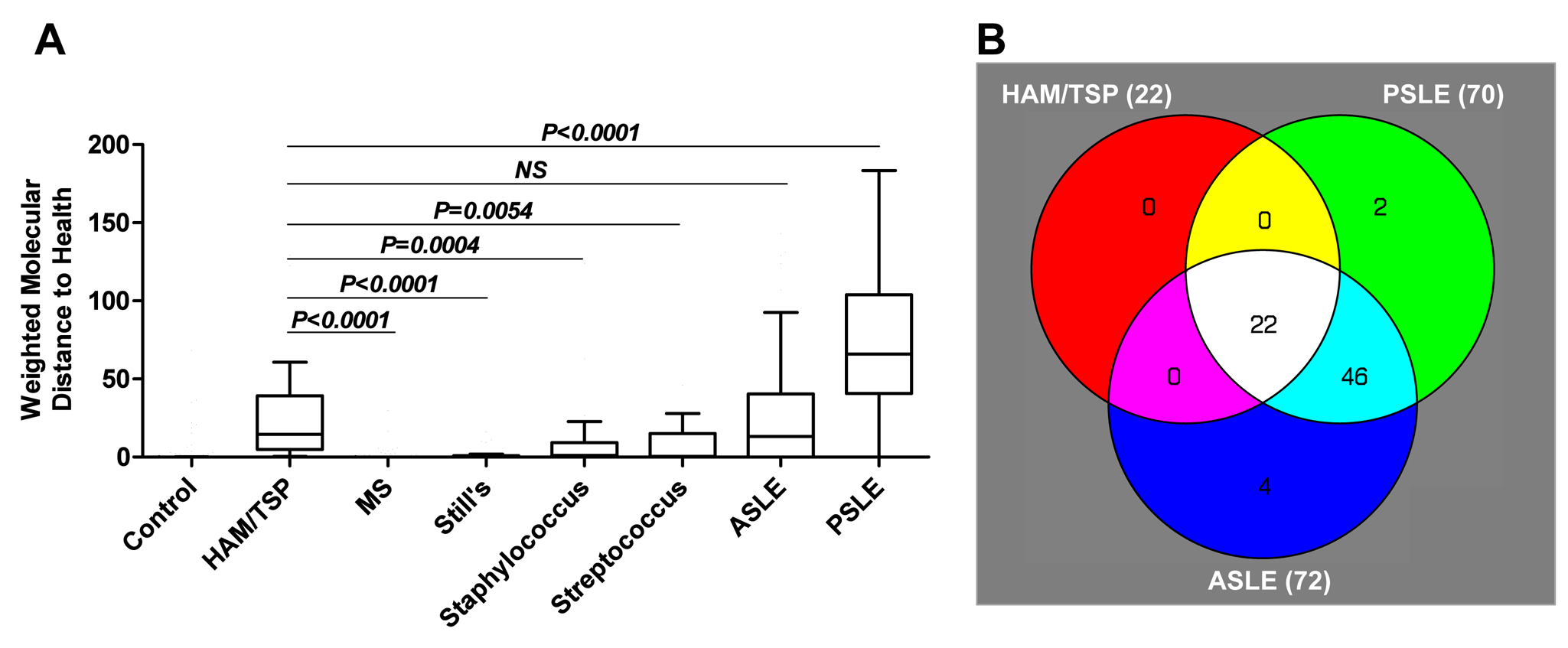

Supplement: Figure S3 — Expression of a distinct subset of IFN-stimulated genes distinguishes HAM/TSP from other diseases. (A) Transcriptional changes for all IFN-stimulated genes comprised in the 80-gene blood transcriptional signature were calculated using the WMDH metric and compared between patients with HAM/TSP (patients n = 20, control n = 17), multiple sclerosis (MS; n = 99, control n = 45), Still's disease (Still's; n = 31, control n = 22), individuals infected with Staphylococcus (n = 40, control n = 12) or group A Streptococcus (n = 23, control n = 12) and patients with adult (ASLE; n = 28, control = 15) or pediatric systemic lupus erythematosus (PSLE; n = 82, control = 18). P-values were calculated using a two-tailed Mann-Whitney test; box plots represent median ± 1.5 IQR. (B) In contrast to patients with SLE, transcriptional changes in patients with HAM/TSP were limited to a small subset of IFN-stimulated genes. Based on list of 76 IFN-stimulated genes in module M3.1, non-parametric group comparisons were performed between patients with HAM/TSP and adult or paediatric systemic lupus erythematosus (ASLE and PSLE). Numbers in brackets depict the number of genes that were significantly different expressed between patients with the disease and their respective healthy controls (two-tailed Mann Whitney test, P<0.05). (TIF) [file ppat.1002480.s003.tif]

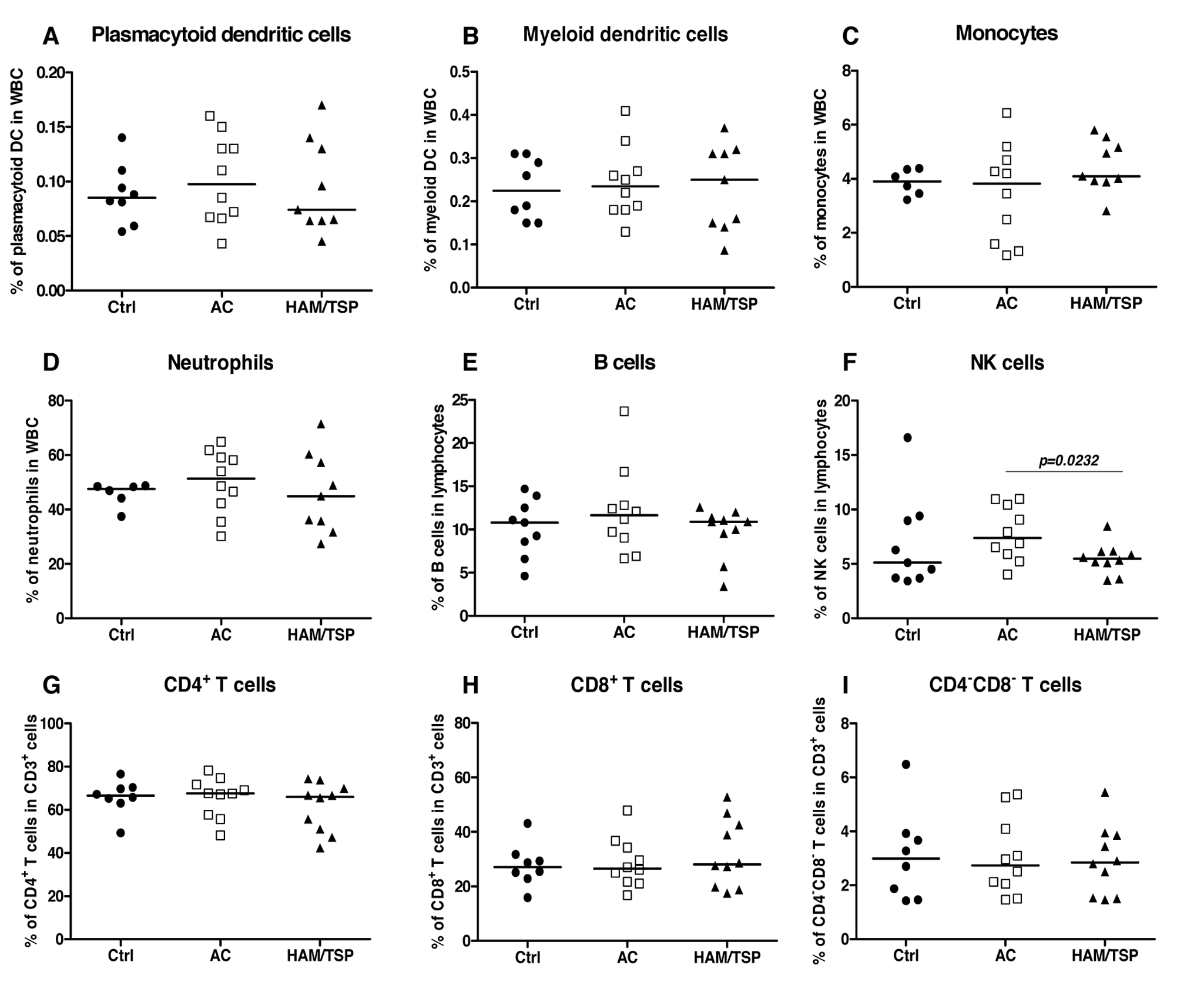

Supplement: Figure S4 — Relative frequencies of peripheral blood cell populations in HTLV-1-positive patients and healthy controls. Cells from heparinised blood were stained with monoclonal antibodies corresponding to well-characterised markers of antigen-presenting and effector cell populations. Following lysis of red blood cells using the BD FACS Lysing solution, the samples were analysed by flow cytometry. (A) plasmacytoid dendritic cells, (B) myeloid dendritic cells, (C) monocytes, (D) neutrophils, (E) B cells, (F) NK cells, (G) CD4+ T cells, (H) CD8+ T cells, (I) CD4−CD8− T cells; WBC = white blood cells. P-values were calculated using a two-tailed Mann-Whitney test; Ctrl: n≤6, AC: n≤9, HAM/TSP: n≤9. (TIF) [file ppat.1002480.s004.tif]

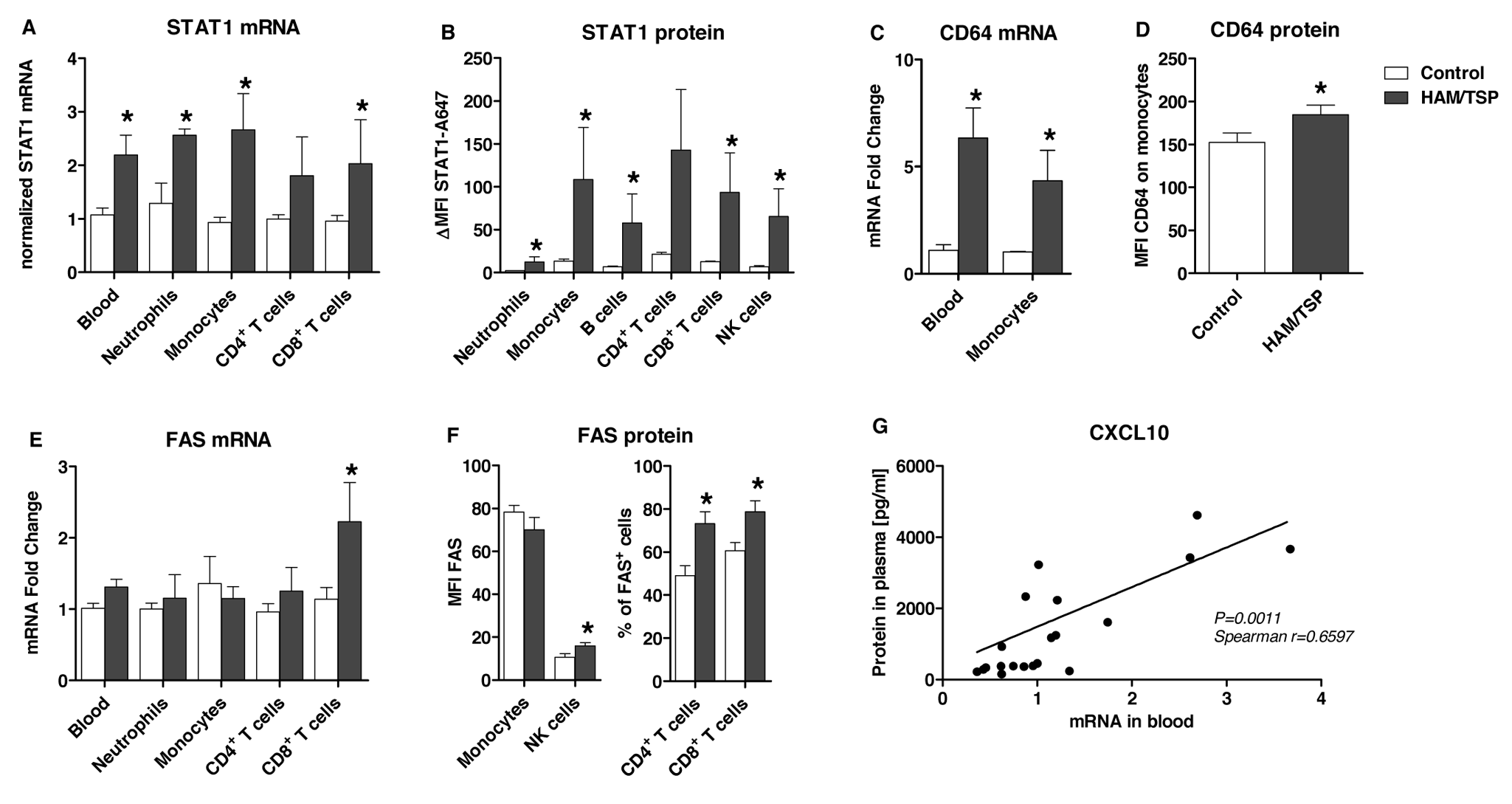

Supplement: Figure S5 — Validation of the IFN-inducible transcriptional signature in HAM/TSP on the protein level. The mRNA and protein levels of the IFN-stimulated genes (A, B) STAT1, (C, D) CD64 and (E, F) FAS were quantified by real-time PCR and flow cytometry on peripheral leukocyte populations. Data represents mean ± SEM; P-values were calculated using a two-tailed Mann-Whitney test with*P<0.05; mRNA: Ctrl: n = 4, HAM/TSP: n = 4; protein: Ctrl: n = 10, HAM/TSP: n = 10. (G) Blood mRNA levels of CXCL10 correlated well with CXCL10 protein levels in plasma (Spearman correlation P-value = 0.0011). AC: n = 13, HAM/TSP: n = 13. (TIF) [file ppat.1002480.s005.tif]

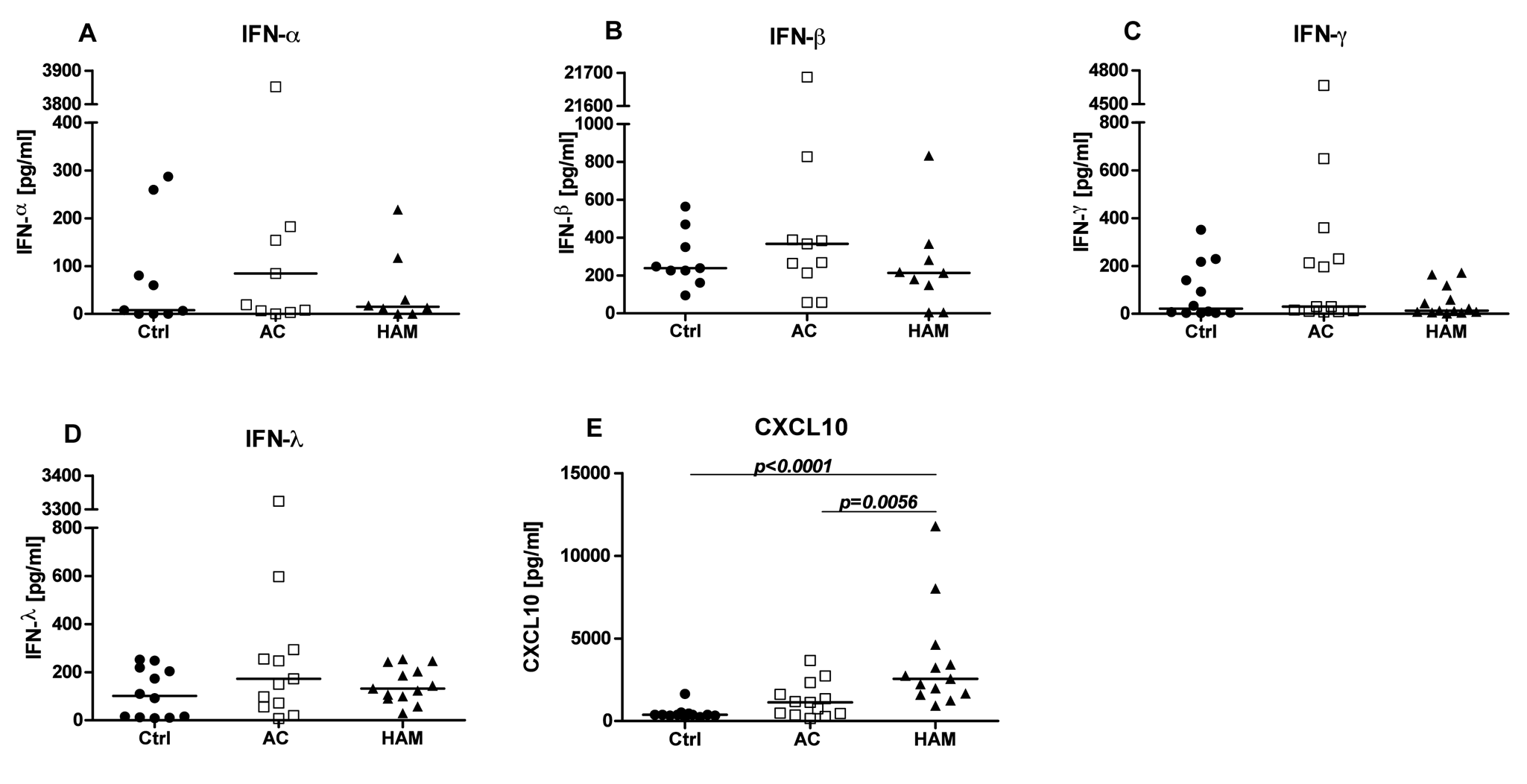

Supplement: Figure S6 — Plasma concentrations of Type I, II and Type III IFNs and CXCL10. Plasma was cleared of blood cells by spin centrifugation and analyzed by luminex for concentrations of (A) IFN- α, (B) IFN-β, (C) IFN-γ, (D) IFN-λ and the IFN-γ-inducible chemokine (E) CXCL10. P-values were calculated using a two-tailed Mann-Whitney test. No Type I and Type III IFN bioactivity was detected in plasma samples (data not shown). Ctrl: n = 12, AC: n = 13, HAM/TSP: n = 13. (TIF) [file ppat.1002480.s006.tif]

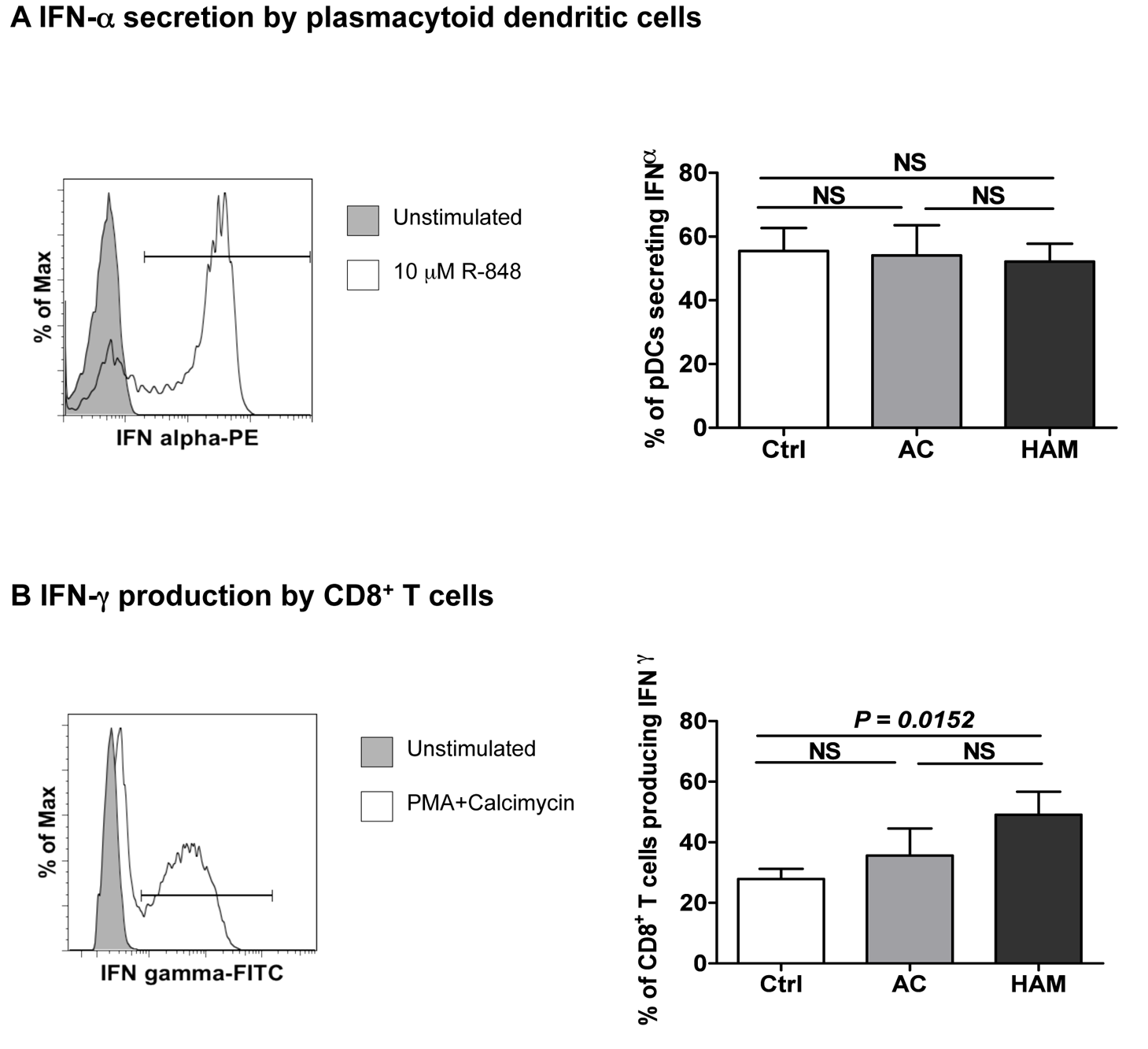

Supplement: Figure S7 — Production of IFN-α and IFN-γ in HTLV-1 carriers. (A) IFN-α secretion by plasmacytoid dendritic cells (pDCs) in whole blood in response to stimulation of TLR7 and TLR8. Fresh blood was stimulated with 10 µM R-848 for 3 h at 37°C. Secreted IFN-α was captured on the surface of pDCs and quantified by flow cytometry. (B) Intracellular production of IFN-γ in CD8+ T cells after PMA/calcimycin stimulation. Fresh blood was stimulated with 10 ng/ml PMA and 0.5 µg/ml calcimycin in the presence of monensin for 4 h at 37°C. Intracellular production of IFN-γ in CD8+ T cells was analyzed by flow cytometry. Histograms are gated on (A) pDCs or (B) CD8+ T cells; unstimulated controls are indicated as grey shaded area, stimulated samples are indicated by a solid black line. Data represents mean ± SEM; P-values were calculated using a two-tailed Mann-Whitney test; Ctrl: n = 6, AC: n = 6, HAM/TSP: n = 6. (TIF) [file ppat.1002480.s007.tif]

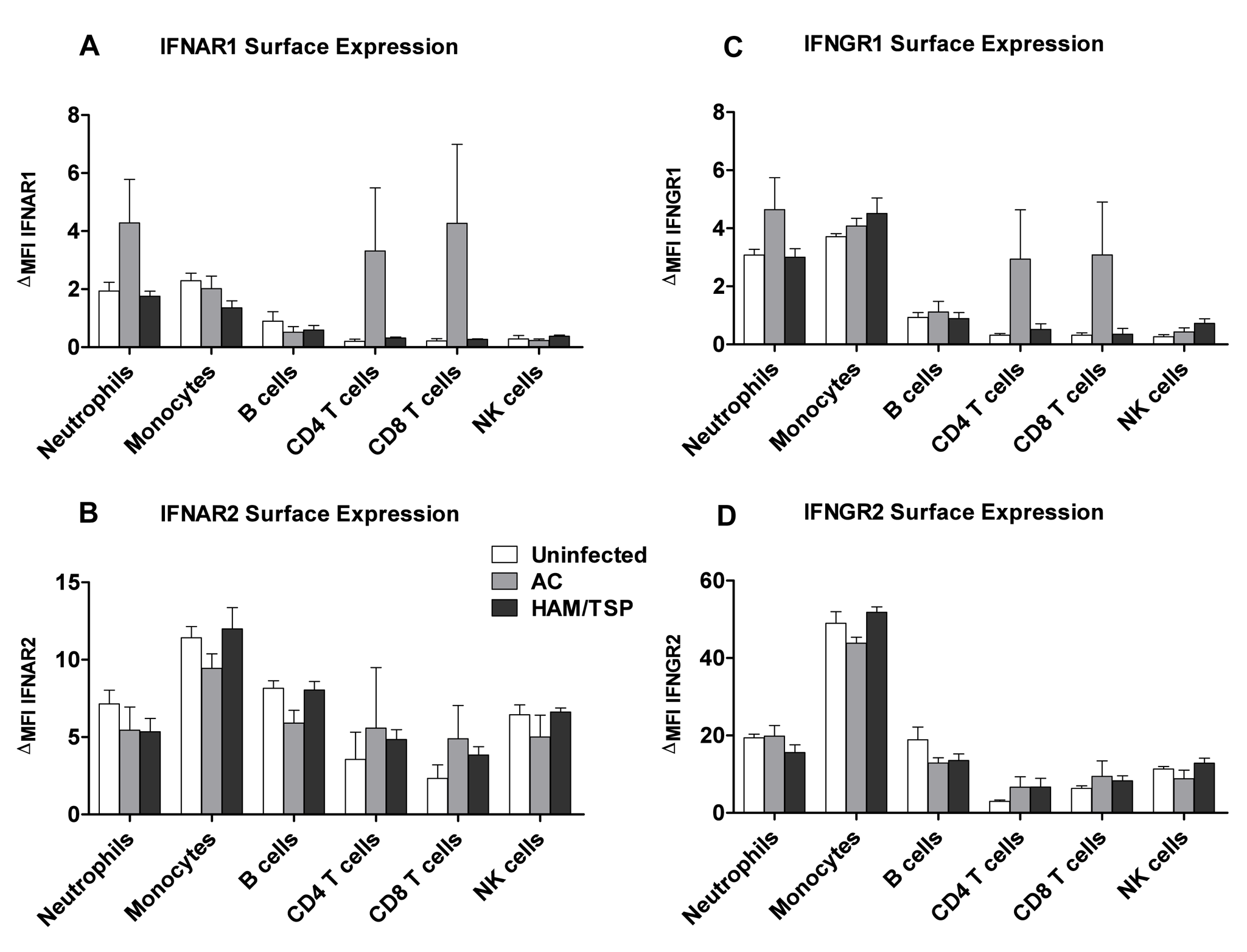

Supplement: Figure S8 — Surface expression of IFN receptors. Heparinised blood samples were analysed by flow cytometry after staining with monoclonal antibodies corresponding to leukocyte populations and (A) IFN-α receptor 1 (IFNAR1), (B) IFN-α receptor 2 (IFNAR2), (C) IFN-γ receptor 1 (IFNGR1) or (D) IFN-γ receptor 2 (IFNGR2). No significant changes in IFN receptor abundance were detected between the study groups. Data represents mean ± SEM; P-values were calculated using a two-tailed Mann-Whitney test; Ctrl: n = 6, AC: n = 5, HAM/TSP: n = 4. (TIF) [file ppat.1002480.s008.tif]

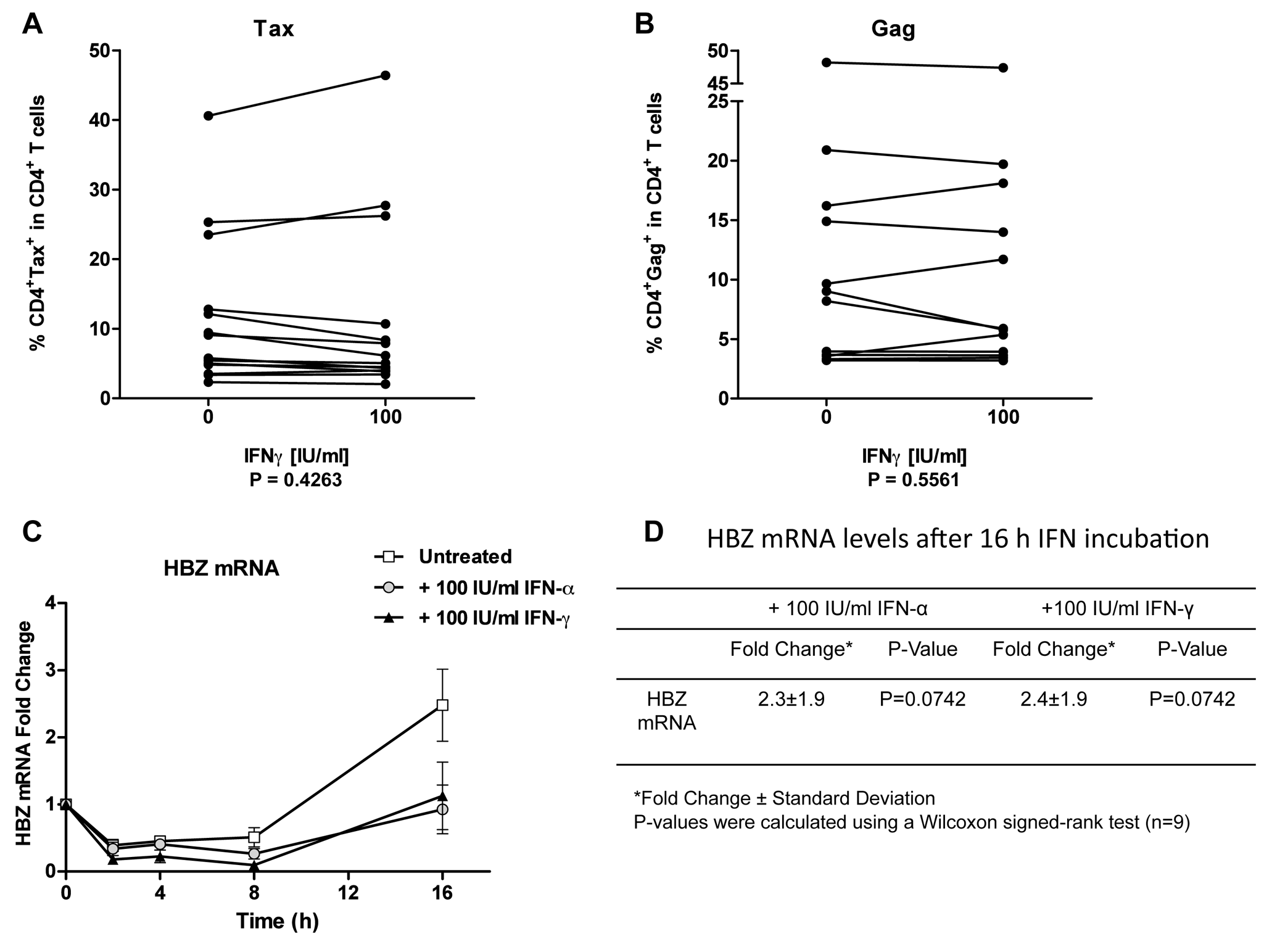

Supplement: Figure S9 — Effect of exogenous IFN on Tax and Gag protein and HBZ mRNA expression. Fresh PBMCs were depleted of CD8+ T cells and incubated for 16 h in the absence or presence of 100 IU/ml IFN-γ (Tax, Gag, HBZ) or IFN-α (HBZ). (A) Tax (P = 0.4263) and (B) Gag (P = 0.5561) protein levels were not altered by IFN-γ treatment as measured by flow cytometry. (C) HBZ mRNA levels were quantified by real-time PCR after 0, 2, 4, 8 and 16 h in response to IFN-α and IFN-γ treatment. Graph depicts mean ± SEM; AC: n = 2, HAM/TSP: n = 2. (D) Table depicts the average HBZ mRNA fold change after 16 h incubation with IFNs. P-values were calculated using a Wilcoxon signed-rank test. AC: n = 2, HAM/TSP: n = 7. (TIF) [file ppat.1002480.s009.tif]
