# Supplementary material for: Systems Biology Approaches Reveal a Specific Interferon-Inducible Signature in HTLV-1 Associated Myelopathy
Source: PLoS Pathog. 2012 Jan 26;8(1):e1002480. doi: 10.1371/journal.ppat.1002480 (PMC3266939; doi:10.1371/journal.ppat.1002480)
Supplement: Table S1 — Demographics characteristics of uninfected and HTLV-1-positive individuals in the (A) microarray training and (B) test set. (DOC) [file ppat.1002480.s010.doc]

**Table S1. Demographics characteristics of uninfected and HTLV-1-positive individuals in the (A) microarray training and (B) test set.**

A Training set

|  | | **Uninfected** | **Asymptomatic Carriers** | | **HAM/TSP** | |
| --- | --- | --- | --- | --- | --- | --- |
|  | |  | **PVL <1% (low)** | **PVL >1% (high)** | |  |
| Number of samples | | 9 | 11 | 9 | 10 | |
| Age (median/quartiles, years) | | 41 (39-43) | 59 (52-63) | 50 (34-53) | 56 (53-68) | |
| PVL (median/quartiles, %) | | -- | 0.3 (0.1-0.4) | 2.6 (1.4-6.2) | 5.9 (3.3-11.2) | |
| Gender | Male | 1 (11%) | 2 (18%) | 1 (11%) | 1 (10%) | |
|  | Female | 8 (89%) | 9 (82%) | 8 (89%) | 9 (90%) | |
| Ethnicity | African | 0 (0%) | 0 (0%) | 2 (22%) | 1 (10%) | |
|  | Afro-Caribbean | 4 (44%) | 7 (63%) | 6 (67%) | 7 (70%) | |
|  | Caucasian | 5 (56%) | 4 (37%) | 1 (11%) | 2 (20%) | |

B Test set

|  | | **Uninfected** | **Asymptomatic Carriers** | | **HAM/TSP** | |
| --- | --- | --- | --- | --- | --- | --- |
|  | |  | **PVL <1% (low)** | **PVL >1% (high)** | |  |
| Number of samples | | 8 | 4 | 13 | 10 | |
| Age (median/quartiles, years) | | 49 (46-71) | 47 (38-62) | 54 (49-62) | 63 (44-74) | |
| PVL (median/quartiles, %) | | -- | 0.6 (0.4-0.9) | 3.9 (3.4-6.7) | 10.1 (6.7-17.8) | |
| Gender | Male | 4 (50%) | 0 (0%) | 0 (0%) | 3 (30%) | |
|  | Female | 4 (50%) | 4 (100%) | 13 (100%) | 7 (70%) | |
| Ethnicity | African | 0 (0%) | 1 (25%) | 0 (0%) | 0 (0%) | |
|  | Afro-Caribbean | 5 (62%) | 2 (50%) | 10 (77%) | 9 (90%) | |
|  | Caucasian | 3 (38%) | 1 (25%) | 3 (23%) | 1 (10%) | |
