# Supplementary material for: Systems Biology Approaches Reveal a Specific Interferon-Inducible Signature in HTLV-1 Associated Myelopathy
Source: PLoS Pathog. 2012 Jan 26;8(1):e1002480. doi: 10.1371/journal.ppat.1002480 (PMC3266939; doi:10.1371/journal.ppat.1002480)
Supplement: Table S2 — List of the 542 transcripts deregulated in HTLV-1 infection. (DOC) [file ppat.1002480.s011.doc]

**Table S2. List of the 542 transcripts deregulated in HTLV-1 infection.**

| **Illumina HT12 v3 ID** | **Gene Symbol** | **Genbank** |
| --- | --- | --- |
| ILMN_1776157 | SEPT4 | NM_080415.1 |
| ILMN_1789627 | SEPT5 | NM_002688.4 |
| ILMN_2061446 | AADACL1 | NM_020792.3 |
| ILMN_2094875 | ABCB1 | NM_000927.3 |
| ILMN_1812070 | ABCB1 | NM_000927.3 |
| ILMN_1677814 | ABCC3 | NM_003786.2 |
| ILMN_2396672 | ABLIM1 | NM_001003407.1 |
| ILMN_1785424 | ABLIM1 | NM_006720.3 |
| ILMN_1656940 | ABLIM3 | NM_014945.2 |
| ILMN_1740265 | ACOT7 | NM_181864.2 |
| ILMN_1784203 | ACRBP | NM_032489.2 |
| ILMN_2148360 | ADAM10 | NM_001110.2 |
| ILMN_1664236 | ADAM28 | NM_021777.3 |
| ILMN_1730504 | AGPAT4 | NM_020133.2 |
| ILMN_2307656 | AGTRAP | NM_001040194.1 |
| ILMN_1752159 | AHNAK | NM_024060.2 |
| ILMN_1681301 | AIM2 | NM_004833.1 |
| ILMN_1670282 | AIRE | NM_000658.1 |
| ILMN_1712530 | AKAP1 | NM_003488.3 |
| ILMN_1713124 | AKR1C3 | NM_003739.4 |
| ILMN_1687757 | AKR1C4 | NM_001818.2 |
| ILMN_1670870 | ALCAM | NM_001627.2 |
| ILMN_1690252 | ALKBH2 | NM_001001655.1 |
| ILMN_1713731 | ALOX12 | NM_000697.2 |
| ILMN_1793894 | ANAPC13 | NM_015391.2 |
| ILMN_2132599 | ANKRD22 | NM_144590.1 |
| ILMN_1799848 | ANKRD22 | NM_144590.2 |
| ILMN_2214278 | ANKRD32 | NM_032290.2 |
| ILMN_1798006 | ANKRD35 | NM_144698.2 |
| ILMN_1711408 | ANXA4 | NM_001153.2 |
| ILMN_2325338 | APOL2 | NM_145637.1 |
| ILMN_1736112 | ARHGAP10 | NM_024605.3 |
| ILMN_1660544 | ARRDC4 | NM_183376.1 |
| ILMN_2184064 | ARRDC4 | NM_183376.1 |
| ILMN_1769013 | ASGR1 | NM_001671.2 |
| ILMN_1815184 | ASPM | NM_018136.3 |
| ILMN_2374865 | ATF3 | NM_001040619.1 |
| ILMN_1779214 | ATM | NM_000051.3 |
| ILMN_1730291 | ATP1B1 | NM_001677.3 |
| ILMN_2407824 | ATP1B1 | NM_001001787.1 |
| ILMN_2376771 | ATR | NM_001184.2 |
| ILMN_2271627 | ATXN2L | NM_007245.2 |
| ILMN_1749081 | AUTS2 | NM_015570.1 |
| ILMN_1724480 | AXIN2 | NM_004655.2 |
| ILMN_1761093 | B3GAT1 | NM_018644.3 |
| ILMN_1669323 | BACE2 | NM_138992.1 |
| ILMN_1690241 | BATF2 | NM_138456.3 |
| ILMN_1763207 | BATF3 | NM_018664.1 |
| ILMN_1704452 | BCL9 | NM_004326.2 |
| ILMN_2351638 | BEX4 | NM_001080425.1 |
| ILMN_1806473 | BEX5 | NM_001012978.1 |
| ILMN_1747650 | BMP6 | NM_001718.4 |
| ILMN_1724658 | BNIP3 | NM_004052.2 |
| ILMN_1697546 | BRCC3 | NM_024332.2 |
| ILMN_1800619 | BRI3BP | NM_080626.5 |
| ILMN_1770161 | BST1 | NM_004334.1 |
| ILMN_1802708 | BTN3A1 | NM_007048.4 |
| ILMN_1749868 | C10ORF38 | NM_001010924.1 |
| ILMN_1790100 | C11ORF82 | NM_145018.2 |
| ILMN_1730523 | C16ORF14 | NM_138418.2 |
| ILMN_1693630 | C16ORF7 | NM_004913.2 |
| ILMN_1702526 | C17ORF48 | NM_020233.4 |
| ILMN_1717793 | C19ORF33 | NM_033520.1 |
| ILMN_1692834 | C1ORF26 | NM_017673.5 |
| ILMN_1796409 | C1QB | NM_000491.3 |
| ILMN_1662640 | C20ORF127 | NM_080757.1 |
| ILMN_1791248 | C20ORF175 | NM_080829.1 |
| ILMN_1785644 | C20ORF29 | NM_018347.1 |
| ILMN_1712386 | C21ORF45 | NM_018944.2 |
| ILMN_2224486 | C3ORF14 | NM_020685.3 |
| ILMN_1672124 | C4ORF18 | NM_016613.4 |
| ILMN_1672605 | C7ORF41 | NM_152793.2 |
| ILMN_1714278 | C9ORF30 | NM_080655.1 |
| ILMN_1762508 | C9ORF72 | NM_145005.3 |
| ILMN_1783598 | CAB39L | NM_001079670.1 |
| ILMN_1676973 | CABP5 | NM_019855.3 |
| ILMN_1671263 | CACNA1H | NM_021098.2 |
| ILMN_1754076 | CACNA2D3 | NM_018398.2 |
| ILMN_1730487 | CALD1 | NM_033140.2 |
| ILMN_1743021 | CAMKK2 | NM_153500.1 |
| ILMN_1792660 | CAMSAP1L1 | NM_203459.1 |
| ILMN_1722158 | CASP5 | NM_004347.1 |
| ILMN_2373763 | CASP7 | NM_033339.3 |
| ILMN_1686920 | CCDC58 | NM_001017928.2 |
| ILMN_2228845 | CCL28 | NM_148672.2 |
| ILMN_2374425 | CCNE1 | NM_001238.1 |
| ILMN_1715131 | CCR7 | NM_001838.2 |
| ILMN_1722622 | CD163 | NM_203416.2 |
| ILMN_2379599 | CD163 | NM_203416.1 |
| ILMN_1733270 | CD163 | NM_004244.4 |
| ILMN_2174805 | CD300LG | NM_145273.2 |
| ILMN_2070940 | CD302 | NM_014880.3 |
| ILMN_1716797 | CD302 | NM_014880.3 |
| ILMN_1792538 | CD7 | NM_006137.6 |
| ILMN_1704730 | CD93 | NM_012072.3 |
| ILMN_2379560 | CDC14B | NM_033331.2 |
| ILMN_1737184 | CDCA7 | NM_031942.4 |
| ILMN_1784602 | CDKN1A | NM_000389.2 |
| ILMN_2371724 | CEACAM1 | NM_001024912.1 |
| ILMN_1795933 | CHMP7 | NM_152272.2 |
| ILMN_1732831 | CHST7 | NM_019886.2 |
| ILMN_1653166 | CLEC10A | NM_006344.2 |
| ILMN_1796423 | CLIC3 | NM_004669.2 |
| ILMN_1682501 | CNOT1 | NM_206999.1 |
| ILMN_1711514 | COCH | NM_004086.1 |
| ILMN_1698621 | COG5 | NM_006348.2 |
| ILMN_1721535 | COG5 | NM_006348.2 |
| ILMN_1726591 | COP1 | NM_052889.2 |
| ILMN_1720484 | CRTAP | NM_006371.3 |
| ILMN_2392189 | CTDSPL | NM_005808.2 |
| ILMN_1665655 | CTDSPL2 | NM_016396.1 |
| ILMN_1792885 | CTSC | NM_001814.2 |
| ILMN_2387224 | CTSE | NM_148964.1 |
| ILMN_1758895 | CTSK | NM_000396.2 |
| ILMN_1744912 | CTTN | NM_005231.2 |
| ILMN_2393712 | CTTN | NM_138565.1 |
| ILMN_1752562 | CXCL5 | NM_002994.3 |
| ILMN_2171384 | CXCL5 | NM_002994.3 |
| ILMN_1779234 | CXCL6 | NM_002993.2 |
| ILMN_1745256 | CXXC5 | NM_016463.5 |
| ILMN_1708303 | CYP4F22 | NM_173483.2 |
| ILMN_1772627 | D4S234E | NM_001040101.1 |
| ILMN_2209671 | DCDC5 | NM_198462.2 |
| ILMN_1805696 | DFFA | NM_213566.1 |
| ILMN_2384181 | DHRS9 | NM_005771.3 |
| ILMN_1733998 | DHRS9 | NM_005771.3 |
| ILMN_1727150 | DHRS9 | NM_199204.1 |
| ILMN_2285618 | DIP2A | NM_015151.2 |
| ILMN_1767651 | DKFZP434B0335 | NM_015395.1 |
| ILMN_1892638 | DKFZP564C152 | AL049980 |
| ILMN_2049184 | DNASE1L3 | NM_004944.2 |
| ILMN_1700822 | DPP3 | NM_005700.3 |
| ILMN_1692535 | DPP4 | NM_001935.3 |
| ILMN_1811328 | DPP7 | NM_013379.2 |
| ILMN_2402640 | DSC1 | NM_004948.2 |
| ILMN_2401873 | DUSP10 | NM_144729.1 |
| ILMN_1690939 | ECGF1 | NM_001953.2 |
| ILMN_1767322 | EDAR | NM_022336.2 |
| ILMN_2371055 | EFNA1 | NM_004428.2 |
| ILMN_1805902 | ELA1 | NM_001971.4 |
| ILMN_1737965 | ELOVL4 | NM_022726.2 |
| ILMN_1780601 | EMR1 | NM_001974.3 |
| ILMN_1664265 | EPHA1 | NM_005232.3 |
| ILMN_2388547 | EPSTI1 | NM_033255.2 |
| ILMN_2352131 | ERBB2 | NM_004448.2 |
| ILMN_1668092 | ESAM | NM_138961.1 |
| ILMN_1788625 | EXOC5 | NM_006544.3 |
| ILMN_2364529 | EZH2 | NM_152998.1 |
| ILMN_1717163 | F13A1 | NM_000129.3 |
| ILMN_2102951 | FAHD2A | NM_016044.1 |
| ILMN_1714418 | FAM101B | NM_182705.2 |
| ILMN_2291455 | FAM102A | NM_001035254.1 |
| ILMN_1788416 | FAM108C1 | NM_021214.1 |
| ILMN_2319077 | FAS | NM_152877.1 |
| ILMN_1808132 | FAS | NM_152872.1 |
| ILMN_1700232 | FBXO30 | NM_032145.4 |
| ILMN_1701455 | FBXO6 | NM_018438.4 |
| ILMN_2176063 | FCGR1A | NM_000566.2 |
| ILMN_2391051 | FCGR1B | NM_001004340.1 |
| ILMN_2261600 | FCGR1B | NM_001017986.1 |
| ILMN_1782015 | FCRLB | NM_001002901.2 |
| ILMN_2370976 | FER1L3 | NM_013451.2 |
| ILMN_1810289 | FER1L3 | NM_133337.1 |
| ILMN_1795865 | FGFRL1 | NM_021923.3 |
| ILMN_2355831 | FHL2 | NM_201555.1 |
| ILMN_1668411 | FHL2 | NM_201557.2 |
| ILMN_2303955 | FKBP1B | NM_054033.1 |
| ILMN_1778444 | FKBP5 | NM_004117.2 |
| ILMN_1733947 | FKBP8 | NM_012181.3 |
| ILMN_1707286 | FLJ22662 | NM_024829.4 |
| ILMN_1693471 | FLJ33790 | NM_001039548.1 |
| ILMN_1687335 | FLNA | NM_001456.2 |
| ILMN_1789400 | FOXD2 | NM_004474.3 |
| ILMN_1682495 | FOXP1 | NM_032682.4 |
| ILMN_1811692 | FTSJ3 | NM_017647.2 |
| ILMN_1724236 | FUT7 | NM_004479.2 |
| ILMN_1781045 | FXYD2 | NM_021603.2 |
| ILMN_1768812 | FXYD6 | NM_022003.1 |
| ILMN_2052208 | GADD45A | NM_001924.2 |
| ILMN_1657475 | GALT | NM_000155.2 |
| ILMN_1794595 | GAMT | NM_000156.4 |
| ILMN_1756469 | GAMT | NM_000156.4 |
| ILMN_1784749 | GAS6 | NM_000820.1 |
| ILMN_1741837 | GBAP | NR_002188.1 |
| ILMN_1701114 | GBP1 | NM_002053.1 |
| ILMN_2148785 | GBP1 | NM_002053.1 |
| ILMN_1725314 | GBP3 | NM_018284.2 |
| ILMN_2114568 | GBP5 | NM_052942.2 |
| ILMN_1725471 | GK | NM_000167.3 |
| ILMN_2217513 | GLB1L | NM_024506.3 |
| ILMN_1782419 | GNG11 | NM_004126.3 |
| ILMN_2401033 | GOSR1 | NM_001007024.1 |
| ILMN_1753575 | GP1BA | NM_000173.4 |
| ILMN_1732269 | GP6 | NM_001083899.1 |
| ILMN_1743290 | GP9 | NM_000174.2 |
| ILMN_2283325 | GPR177 | NM_024911.4 |
| ILMN_1805973 | GPR19 | NM_006143.1 |
| ILMN_1701947 | GPR34 | NM_005300.3 |
| ILMN_1778143 | GRAP2 | NM_004810.2 |
| ILMN_1713162 | GSTM2 | NM_000848.2 |
| ILMN_2157957 | GTF2H1 | NM_005316.2 |
| ILMN_1731233 | GZMH | NM_033423.3 |
| ILMN_1694268 | HES6 | NM_018645.3 |
| ILMN_1686478 | HIST1H2AG | NM_021064.3 |
| ILMN_1651496 | HIST1H2BD | NM_138720.1 |
| ILMN_1758623 | HIST1H2BD | NM_138720.1 |
| ILMN_1732071 | HIST2H2BE | NM_003528.2 |
| ILMN_2115340 | HIST2H4A | NM_003548.2 |
| ILMN_2066060 | HLA-DRB6 | NR_001298.1 |
| ILMN_1720059 | HMBOX1 | NM_024567.2 |
| ILMN_1705984 | HNMT | NM_006895.2 |
| ILMN_1671486 | HOMER2 | NM_199331.2 |
| ILMN_1741406 | HOOK1 | NM_015888.4 |
| ILMN_1768101 | HOXB6 | NM_018952.4 |
| ILMN_1867588 | HS.103173 | BX100504 |
| ILMN_1851599 | HS.130245 | BX110640 |
| ILMN_1911047 | HS.14706 | AY358510 |
| ILMN_1915777 | HS.202313 | BQ189294 |
| ILMN_1914343 | HS.211821 | BX090817 |
| ILMN_1836309 | HS.326560 | XM_498568 |
| ILMN_1915076 | HS.355933 | AK123915 |
| ILMN_1864558 | HS.382309 | BC034632 |
| ILMN_1906423 | HS.390407 | BX097705 |
| ILMN_1847870 | HS.445121 | BM545878 |
| ILMN_1866389 | HS.446266 | BQ182905 |
| ILMN_1868047 | HS.537779 | BP398340 |
| ILMN_1865639 | HS.548213 | AI630987 |
| ILMN_1859863 | HS.555181 | BM458075 |
| ILMN_1856991 | HS.557339 | AA080911 |
| ILMN_1818632 | HS.561844 | BX101194 |
| ILMN_1886769 | HS.572538 | CF454561 |
| ILMN_1911428 | HS.573549 | BG190571 |
| ILMN_1851535 | HS.578214 | DA957648 |
| ILMN_1842582 | HS.579530 | CD522953 |
| ILMN_1832508 | HS.583642 | BG190619 |
| ILMN_1910908 | HS.72010 | BG205162 |
| ILMN_1654566 | HSPA1L | NM_005527.3 |
| ILMN_1901198 | HSPC157 | BC071749 |
| ILMN_1671913 | HTF9C | NM_022727.4 |
| ILMN_1732296 | ID3 | NM_002167.2 |
| ILMN_1707695 | IFIT1 | NM_001548.3 |
| ILMN_1739428 | IFIT2 | NM_001547.4 |
| ILMN_1701789 | IFIT3 | NM_001031683.1 |
| ILMN_1664543 | IFIT3 | NM_001031683.1 |
| ILMN_1805750 | IFITM3 | NM_021034.2 |
| ILMN_1807423 | IGF2BP3 | NM_006547.2 |
| ILMN_2369221 | IL15 | NM_172174.1 |
| ILMN_1724181 | IL15 | NM_000585.2 |
| ILMN_1813572 | IL16 | NM_172217.2 |
| ILMN_1715603 | IL23A | NM_016584.2 |
| ILMN_1683774 | IL2RA | NM_000417.1 |
| ILMN_2342579 | IL7R | NM_002185.2 |
| ILMN_1691507 | IL8RBP | XR_001023.1 |
| ILMN_1707979 | INCA | NM_001007232.1 |
| ILMN_2054019 | ISG15 | NM_005101.1 |
| ILMN_1747934 | ISYNA1 | NM_016368.3 |
| ILMN_1733324 | ITGB3 | NM_000212.2 |
| ILMN_1668374 | ITGB5 | NM_002213.3 |
| ILMN_1661519 | KIAA0408 | NM_014702.3 |
| ILMN_1690139 | KIAA0748 | XM_934138.1 |
| ILMN_2058975 | KIAA0776 | NM_015323.2 |
| ILMN_2143155 | KIF11 | NM_004523.2 |
| ILMN_1803018 | KIFC2 | NM_145754.2 |
| ILMN_1693207 | KIR2DL4 | NM_002255.5 |
| ILMN_2229379 | KIT | NM_000222.1 |
| ILMN_2386790 | KLRC3 | NM_002261.2 |
| ILMN_1672120 | KRT73 | NM_175068.2 |
| ILMN_1660635 | LACTB2 | NM_016027.1 |
| ILMN_1683792 | LAP3 | NM_015907.2 |
| ILMN_1763537 | LCTL | NM_207338.2 |
| ILMN_1809040 | LDLRAP1 | NM_015627.2 |
| ILMN_1679185 | LEF1 | NM_016269.2 |
| ILMN_1698019 | LGMN | NM_001008530.1 |
| ILMN_1747744 | LHFPL2 | NM_005779.1 |
| ILMN_2355953 | LILRB4 | NM_001081438.1 |
| ILMN_2184708 | LIN7C | NM_018362.2 |
| ILMN_2343105 | LIPT1 | NM_015929.2 |
| ILMN_1671372 | LOC137107 | XM_940214.1 |
| ILMN_1716468 | LOC152195 | NM_194289.1 |
| ILMN_1798557 | LOC202134 | XM_371783.3 |
| ILMN_1709326 | LOC23117 | XM_933864.1 |
| ILMN_2089752 | LOC285016 | NM_001002919.1 |
| ILMN_1915188 | LOC286440 | AK123807 |
| ILMN_1734149 | LOC388122 | XM_370865.4 |
| ILMN_1782487 | LOC400759 | NR_003133.1 |
| ILMN_1761281 | LOC441019 | XM_498969.2 |
| ILMN_1719579 | LOC441081 | XR_017029.1 |
| ILMN_1707904 | LOC641518 | XR_017788.1 |
| ILMN_1657996 | LOC642035 | XM_936105.1 |
| ILMN_1690970 | LOC642233 | XM_942795.1 |
| ILMN_1678568 | LOC643913 | XM_931913.1 |
| ILMN_1701753 | LOC644063 | XR_016547.1 |
| ILMN_1710458 | LOC646990 | XM_929962.1 |
| ILMN_1672687 | LOC648099 | XM_937154.1 |
| ILMN_1689876 | LOC648189 | XM_937239.2 |
| ILMN_1682968 | LOC648196 | XM_937246.1 |
| ILMN_1711087 | LOC648526 | XM_937579.1 |
| ILMN_1804601 | LOC649923 | XM_939003.1 |
| ILMN_1664398 | LOC651621 | XM_940809.1 |
| ILMN_1733983 | LOC653316 | XM_933119.1 |
| ILMN_1813950 | LOC653486 | XM_927639.1 |
| ILMN_1719149 | LOC653663 | XM_371511.4 |
| ILMN_1652459 | LOC654161 | XM_944884.1 |
| ILMN_1692072 | LOC728006 | XM_001128698.1 |
| ILMN_1785919 | LOC728229 | XM_001128450.1 |
| ILMN_1654389 | LOC728744 | XM_001128342.1 |
| ILMN_1704014 | LOC730256 | XM_001134398.1 |
| ILMN_1728999 | LOC730273 | XM_001124203.1 |
| ILMN_1651610 | LOC730525 | XM_001126202.1 |
| ILMN_1732278 | LOC730996 | XM_001128017.1 |
| ILMN_1703102 | LOC731777 | XR_015950.1 |
| ILMN_1701441 | LPAR1 | NM_057159.2 |
| ILMN_1796335 | LPCAT2 | NM_017839.3 |
| ILMN_1813175 | LPHN1 | NM_014921.3 |
| ILMN_2128795 | LRIG1 | NM_015541.2 |
| ILMN_1718633 | LRP5L | NM_182492.1 |
| ILMN_2048591 | LRRN3 | NM_018334.3 |
| ILMN_1773650 | LRRN3 | NM_001099660.1 |
| ILMN_1795464 | LTA | NM_000595.2 |
| ILMN_1684984 | MAGED2 | NM_177433.1 |
| ILMN_1713807 | MAN1C1 | NM_020379.2 |
| ILMN_2303170 | MBNL3 | NM_133486.1 |
| ILMN_1660462 | MCOLN2 | NM_153259.2 |
| ILMN_1705685 | MEIS1 | NM_002398.2 |
| ILMN_1688775 | METRNL | XM_941466.2 |
| ILMN_1815190 | METTL1 | NM_005371.4 |
| ILMN_1756071 | MFGE8 | NM_005928.1 |
| ILMN_1657708 | MGLL | NM_007283.5 |
| ILMN_1738589 | MGLL | NM_007283.5 |
| ILMN_1781952 | MGST1 | NM_020300.3 |
| ILMN_1864900 | MIAT | NR_003491.1 |
| ILMN_2344988 | MLL5 | NM_182931.2 |
| ILMN_1803005 | MMACHC | NM_015506.2 |
| ILMN_2399016 | MMP28 | NM_001032278.1 |
| ILMN_1717046 | MOBKL2B | NM_024761.3 |
| ILMN_2247988 | MOCS1 | NM_001075098.1 |
| ILMN_1721337 | MRPS18B | NM_014046.2 |
| ILMN_1741404 | MSC | NM_005098.3 |
| ILMN_1691156 | MT1A | NM_005946.2 |
| ILMN_2173611 | MT1E | NM_175617.3 |
| ILMN_1715401 | MT1G | NM_005950.1 |
| ILMN_1686664 | MT2A | NM_005953.2 |
| ILMN_2136089 | MTE | NM_175621.2 |
| ILMN_1756992 | MUC1 | NM_001044391.1 |
| ILMN_1680618 | MYC | NM_002467.3 |
| ILMN_1738523 | MYD88 | NM_002468.3 |
| ILMN_1688417 | MYL2 | NM_000432.1 |
| ILMN_1675062 | MYL9 | NM_006097.3 |
| ILMN_1691476 | MYLK | NM_053032.2 |
| ILMN_2364768 | MYLK | NM_053026.3 |
| ILMN_2315569 | N6AMT1 | NM_182749.2 |
| ILMN_1691264 | NAT8B | NM_016347.2 |
| ILMN_1676289 | NCAM1 | NM_000615.5 |
| ILMN_1751444 | NCAPG | NM_022346.3 |
| ILMN_2335198 | NCOA1 | NM_147233.2 |
| ILMN_2044471 | NCR3 | NM_147130.1 |
| ILMN_1677396 | NDFIP2 | NM_019080.1 |
| ILMN_1758548 | NEK7 | NM_133494.1 |
| ILMN_1758311 | NET1 | NM_001047160.1 |
| ILMN_1783276 | NEXN | NM_144573.3 |
| ILMN_1778991 | NFIB | NM_005596.2 |
| ILMN_2310896 | NLRP3 | NM_004895.3 |
| ILMN_2347592 | NMB | NM_021077.3 |
| ILMN_1657884 | NME2 | NM_002512.2 |
| ILMN_1692413 | NMNAT1 | NM_022787.2 |
| ILMN_1684210 | NPAL3 | NM_020448.3 |
| ILMN_1704206 | NPSR1 | NM_207172.1 |
| ILMN_1661777 | NR3C2 | NM_000901.2 |
| ILMN_2210934 | NR3C2 | NM_000901.1 |
| ILMN_1705686 | NRGN | NM_006176.1 |
| ILMN_1769734 | NT5C3 | NM_001002010.1 |
| ILMN_2410826 | OAS1 | NM_001032409.1 |
| ILMN_1745397 | OAS3 | NM_006187.2 |
| ILMN_1681721 | OASL | NM_003733.2 |
| ILMN_1674811 | OASL | NM_198213.1 |
| ILMN_2382121 | OCRL | NM_001587.3 |
| ILMN_1671288 | ODF2 | NM_002540.3 |
| ILMN_1742025 | OLFM1 | NM_014279.4 |
| ILMN_2045975 | ORMDL1 | NM_016467.3 |
| ILMN_1765061 | OXER1 | NM_148962.4 |
| ILMN_2342835 | P2RY14 | NM_014879.3 |
| ILMN_1723535 | P2RY2 | NM_176071.1 |
| ILMN_1718558 | PARP12 | NM_022750.2 |
| ILMN_2053527 | PARP9 | NM_031458.1 |
| ILMN_1731224 | PARP9 | NM_031458.1 |
| ILMN_1701644 | PCDHGB6 | NM_018926.2 |
| ILMN_1767934 | PCSK5 | NM_006200.2 |
| ILMN_2113535 | PCYOX1 | NM_016297.2 |
| ILMN_1810836 | PDE5A | NM_001083.3 |
| ILMN_2306540 | PDE9A | NM_001001567.1 |
| ILMN_2394305 | PDGFB | NM_033016.1 |
| ILMN_2376859 | PDGFD | NM_025208.4 |
| ILMN_1803094 | PDGFD | NM_033135.3 |
| ILMN_1684982 | PDK4 | NM_002612.3 |
| ILMN_1660232 | PEX5 | NM_000319.3 |
| ILMN_1687978 | PHLDA1 | NM_007350.3 |
| ILMN_1766264 | PI16 | NM_153370.2 |
| ILMN_1671891 | PID1 | NM_017933.3 |
| ILMN_2117323 | PIK3C2B | NM_002646.2 |
| ILMN_1687896 | PIK3C3 | NM_002647.2 |
| ILMN_2391400 | PITX2 | NM_153427.1 |
| ILMN_1720034 | PKHD1 | NM_138694.3 |
| ILMN_2406169 | PKIG | NM_007066.3 |
| ILMN_1810191 | PLA2G4C | NM_003706.1 |
| ILMN_1696270 | PLAG1 | NM_002655.1 |
| ILMN_1718852 | PLCL1 | NM_006226.1 |
| ILMN_2206953 | PLCL1 | NM_006226.1 |
| ILMN_2394250 | PLEKHA1 | NM_021622.3 |
| ILMN_1770850 | PNMA1 | NM_006029.4 |
| ILMN_1751773 | POLD3 | NM_006591.1 |
| ILMN_1690077 | PPM1A | NM_177951.1 |
| ILMN_1713846 | PPM1H | NM_020700.1 |
| ILMN_1761968 | PPP1R14A | NM_033256.1 |
| ILMN_2044226 | PPP3CA | NM_000944.2 |
| ILMN_1809818 | PRCC | NM_005973.4 |
| ILMN_1725188 | PRKCI | NM_002740.5 |
| ILMN_2334121 | PRKDC | NM_006904.6 |
| ILMN_1677509 | PRR7 | NM_030567.3 |
| ILMN_1658243 | PSD3 | NM_206909.2 |
| ILMN_1798233 | PSMB9 | NM_002800.4 |
| ILMN_1713058 | PSTPIP2 | NM_024430.2 |
| ILMN_2051867 | PTCD2 | NM_024754.3 |
| ILMN_1763723 | PTDSS2 | NM_030783.1 |
| ILMN_2339835 | PTGS1 | NM_080591.1 |
| ILMN_1721046 | PTMS | NM_002824.4 |
| ILMN_1744146 | PTTG2 | NM_006607.2 |
| ILMN_2346997 | RAB23 | NM_016277.3 |
| ILMN_1837428 | RAB27B | AF131784 |
| ILMN_2221006 | RAD21 | NM_006265.1 |
| ILMN_2398474 | RAP1B | NM_015646.4 |
| ILMN_2120210 | RCAN2 | NM_005822.2 |
| ILMN_1773395 | RDH5 | NM_002905.2 |
| ILMN_1751886 | REC8 | NM_005132.2 |
| ILMN_2317751 | REC8 | NM_005132.2 |
| ILMN_1757537 | RGAG4 | NM_001024455.2 |
| ILMN_1746138 | RLTPR | NM_001013838.1 |
| ILMN_1706266 | RNF157 | NM_052916.1 |
| ILMN_1792389 | RNF165 | NM_152470.2 |
| ILMN_2357777 | RNF214 | NM_207343.2 |
| ILMN_1734366 | RORC | NM_001001523.1 |
| ILMN_1771126 | RORC | NM_001001523.1 |
| ILMN_1665425 | RPRM | NM_019845.2 |
| ILMN_2411781 | RYR1 | NM_001042723.1 |
| ILMN_1740842 | SALL2 | NM_005407.1 |
| ILMN_1658821 | SAMD1 | NM_138352.1 |
| ILMN_1799467 | SAMD9L | NM_152703.2 |
| ILMN_1700896 | SAP30 | NM_003864.3 |
| ILMN_1655663 | SC65 | NM_006455.2 |
| ILMN_2340908 | SCML1 | NM_001037540.1 |
| ILMN_1701621 | SCO2 | NM_005138.1 |
| ILMN_1756439 | SCRN1 | NM_014766.3 |
| ILMN_1715991 | SDPR | NM_004657.4 |
| ILMN_1742947 | SEC14L4 | NM_174977.2 |
| ILMN_1687268 | SEC14L5 | NM_014692.1 |
| ILMN_1655595 | SERPINE2 | NM_006216.2 |
| ILMN_1670305 | SERPING1 | NM_001032295.1 |
| ILMN_2205935 | SFXN1 | NM_022754.4 |
| ILMN_1701237 | SH2D1B | NM_053282.4 |
| ILMN_1794780 | SHARPIN | NM_030974.2 |
| ILMN_2380566 | SIAH1 | NM_001006610.1 |
| ILMN_1782938 | SLC16A10 | NM_018593.3 |
| ILMN_1729691 | SLC16A6 | NM_004694.3 |
| ILMN_1752639 | SLC25A24 | NM_013386.3 |
| ILMN_1659894 | SLC25A26 | NM_001009938.1 |
| ILMN_2350801 | SLC25A29 | NM_001039355.1 |
| ILMN_1767717 | SLC2A11 | NM_001024939.1 |
| ILMN_2067852 | SLC30A1 | NM_021194.2 |
| ILMN_2203896 | SMAD7 | NM_005904.2 |
| ILMN_2408987 | SMARCD3 | NM_001003802.1 |
| ILMN_1690262 | SNAI3 | NM_178310.1 |
| ILMN_1682354 | SNORD33 | NR_000020.1 |
| ILMN_1740180 | SNX3 | NM_003795.3 |
| ILMN_1707077 | SORT1 | NM_002959.4 |
| ILMN_1789244 | SOX8 | NM_014587.2 |
| ILMN_2367681 | SPAG1 | NM_003114.3 |
| ILMN_2381476 | SPG3A | NM_181598.2 |
| ILMN_1729987 | SRC | NM_198291.1 |
| ILMN_2328986 | SREBF1 | NM_004176.3 |
| ILMN_1726626 | SSR2 | XM_945427.1 |
| ILMN_1699887 | ST14 | NM_021978.2 |
| ILMN_1781135 | STAM2 | NM_005843.3 |
| ILMN_1777325 | STAT1 | NM_007315.2 |
| ILMN_1690105 | STAT1 | NM_007315.2 |
| ILMN_1691364 | STAT1 | NM_139266.1 |
| ILMN_2215119 | SYNJ2 | NM_003898.2 |
| ILMN_1719599 | SYTL4 | NM_080737.1 |
| ILMN_1739001 | TACSTD2 | NM_002353.1 |
| ILMN_1668351 | TAF6 | NM_139315.1 |
| ILMN_1688098 | TBC1D4 | NM_014832.2 |
| ILMN_1703891 | TBC1D9 | NM_015130.2 |
| ILMN_1726928 | TCEA3 | NM_003196.1 |
| ILMN_1676470 | TCF7 | NM_213648.1 |
| ILMN_2367141 | TCF7 | NM_201632.1 |
| ILMN_1740572 | TCN2 | NM_000355.2 |
| ILMN_1685124 | TCTN1 | NM_001082538.1 |
| ILMN_2389876 | TGFB1I1 | NM_015927.3 |
| ILMN_1686116 | THBS1 | NM_003246.2 |
| ILMN_1659610 | TJP3 | NM_014428.1 |
| ILMN_1766814 | TK2 | NM_004614.3 |
| ILMN_1674009 | TKTL1 | NM_012253.2 |
| ILMN_1814917 | TLE2 | NM_003260.3 |
| ILMN_1736585 | TMED10 | NM_006827.5 |
| ILMN_2067607 | TMEM106B | NM_018374.2 |
| ILMN_2194229 | TMEM128 | NM_032927.2 |
| ILMN_1663033 | TMEM129 | NM_138385.2 |
| ILMN_1792455 | TMEM158 | NM_015444.2 |
| ILMN_1773935 | TMEM165 | NM_018475.2 |
| ILMN_1725387 | TMEM200A | NM_052913.2 |
| ILMN_1785732 | TNFAIP6 | NM_007115.2 |
| ILMN_2299661 | TNFRSF25 | NM_001039664.1 |
| ILMN_1765109 | TNFRSF25 | NM_148973.1 |
| ILMN_2399190 | TNFSF13 | NM_172087.1 |
| ILMN_1784264 | TNFSF13 | NM_172088.1 |
| ILMN_2168933 | TNIP2 | NM_024309.2 |
| ILMN_1653180 | TPM4 | NM_003290.1 |
| ILMN_1843100 | TRA@ | AY375451 |
| ILMN_1690783 | TREML1 | NM_178174.2 |
| ILMN_1770865 | TRIM46 | NM_025058.3 |
| ILMN_1719254 | TRIM9 | NM_015163.4 |
| ILMN_2352380 | TRPM2 | NM_001001188.3 |
| ILMN_1787567 | TSC22D1 | NM_006022.2 |
| ILMN_1692177 | TSC22D1 | NM_006022.2 |
| ILMN_1729453 | TSPAN9 | NM_006675.3 |
| ILMN_1710280 | TUBB1 | NM_030773.2 |
| ILMN_1741143 | TXK | NM_003328.2 |
| ILMN_1670764 | USP25 | NM_013396.3 |
| ILMN_1809467 | VAMP5 | NM_006634.2 |
| ILMN_1687301 | VCAN | NM_004385.2 |
| ILMN_1795429 | VCL | NM_014000.2 |
| ILMN_2413527 | VCL | NM_003373.3 |
| ILMN_1727271 | WARS | NM_173701.1 |
| ILMN_2337655 | WARS | NM_004184.3 |
| ILMN_1805863 | WDR81 | NM_152348.1 |
| ILMN_1734544 | WTAP | NM_004906.3 |
| ILMN_1742618 | XAF1 | NM_199139.1 |
| ILMN_2188374 | XPOT | NM_007235.3 |
| ILMN_1743456 | ZCCHC14 | NM_015144.2 |
| ILMN_1663858 | ZNF286A | NM_020652.1 |
| ILMN_1901934 | ZNF33B | BI258188 |
| ILMN_1658094 | ZNF365 | NM_014951.2 |
| ILMN_1654946 | ZSCAN18 | NM_023926.3 |
| ILMN_1789410 | ZSCAN21 | NM_145914.2 |
