# Supplementary material for: Systems Biology Approaches Reveal a Specific Interferon-Inducible Signature in HTLV-1 Associated Myelopathy
Source: PLoS Pathog. 2012 Jan 26;8(1):e1002480. doi: 10.1371/journal.ppat.1002480 (PMC3266939; doi:10.1371/journal.ppat.1002480)
Supplement: Table S3 — List of 80 transcripts deregulated in HAM/TSP. (DOC) [file ppat.1002480.s012.doc]

**Table S3. List of 80 transcripts deregulated in HAM/TSP.**

| **Illumina HT12 v3 ID** | **Gene Symbol** | **Genbank** |
| --- | --- | --- |
| ILMN_2094875 | ABCB1 | NM_000927.3 |
| ILMN_1812070 | ABCB1 | NM_000927.3 |
| ILMN_1670282 | AIRE | NM_000658.1 |
| ILMN_1687757 | AKR1C4 | NM_001818.2 |
| ILMN_1799848 | ANKRD22 | NM_144590.2 |
| ILMN_2132599 | ANKRD22 | NM_144590.1 |
| ILMN_2184064 | ARRDC4 | NM_183376.1 |
| ILMN_1749081 | AUTS2 | NM_015570.1 |
| ILMN_1669323 | BACE2 | NM_138992.1 |
| ILMN_1802708 | BTN3A1 | NM_007048.4 |
| ILMN_1796409 | C1QB | NM_000491.3 |
| ILMN_1722158 | CASP5 | NM_004347.1 |
| ILMN_2228845 | CCL28 | NM_148672.2 |
| ILMN_1732831 | CHST7 | NM_019886.2 |
| ILMN_1711514 | COCH | NM_004086.1 |
| ILMN_2387224 | CTSE | NM_148964.1 |
| ILMN_1745256 | CXXC5 | NM_016463.5 |
| ILMN_1772627 | D4S234E | NM_001040101.1 |
| ILMN_1805696 | DFFA | NM_213566.1 |
| ILMN_1727150 | DHRS9 | NM_199204.1 |
| ILMN_1892638 | DKFZP564C152 | AL049980 |
| ILMN_2049184 | DNASE1L3 | NM_004944.2 |
| ILMN_1737965 | ELOVL4 | NM_022726.2 |
| ILMN_2388547 | EPSTI1 | NM_033255.2 |
| ILMN_1788416 | FAM108C1 | NM_021214.1 |
| ILMN_1808132 | FAS | NM_152872.1 |
| ILMN_2176063 | FCGR1A | NM_000566.2 |
| ILMN_2391051 | FCGR1B | NM_001004340.1 |
| ILMN_2261600 | FCGR1B | NM_001017986.1 |
| ILMN_1810289 | FER1L3 | NM_133337.1 |
| ILMN_2148785 | GBP1 | NM_002053.1 |
| ILMN_1701114 | GBP1 | NM_002053.1 |
| ILMN_2114568 | GBP5 | NM_052942.2 |
| ILMN_1725471 | GK | NM_000167.3 |
| ILMN_1851599 | HS.130245 | BX110640 |
| ILMN_1915777 | HS.202313 | BQ189294 |
| ILMN_1914343 | HS.211821 | BX090817 |
| ILMN_1910908 | HS.72010 | BG205162 |
| ILMN_1654566 | HSPA1L | NM_005527.3 |
| ILMN_1707695 | IFIT1 | NM_001548.3 |
| ILMN_1701789 | IFIT3 | NM_001031683.1 |
| ILMN_1664543 | IFIT3 | NM_001031683.1 |
| ILMN_1805750 | IFITM3 | NM_021034.2 |
| ILMN_1707979 | INCA | NM_001007232.1 |
| ILMN_1690139 | KIAA0748 | XM_934138.1 |
| ILMN_1709326 | LOC23117 | XM_933864.1 |
| ILMN_2089752 | LOC285016 | NM_001002919.1 |
| ILMN_1782487 | LOC400759 | NR_003133.1 |
| ILMN_1804601 | LOC649923 | XM_939003.1 |
| ILMN_1652459 | LOC654161 | XM_944884.1 |
| ILMN_1654389 | LOC728744 | XM_001128342.1 |
| ILMN_1676289 | NCAM1 | NM_000615.5 |
| ILMN_1657884 | NME2 | NM_002512.2 |
| ILMN_2342835 | P2RY14 | NM_014879.3 |
| ILMN_1718558 | PARP12 | NM_022750.2 |
| ILMN_1731224 | PARP9 | NM_031458.1 |
| ILMN_2053527 | PARP9 | NM_031458.1 |
| ILMN_1767934 | PCSK5 | NM_006200.2 |
| ILMN_2206953 | PLCL1 | NM_006226.1 |
| ILMN_2394250 | PLEKHA1 | NM_021622.3 |
| ILMN_1658243 | PSD3 | NM_206909.2 |
| ILMN_2120210 | RCAN2 | NM_005822.2 |
| ILMN_1751886 | REC8 | NM_005132.2 |
| ILMN_2317751 | REC8 | NM_005132.2 |
| ILMN_1792389 | RNF165 | NM_152470.2 |
| ILMN_1734366 | RORC | NM_001001523.1 |
| ILMN_1771126 | RORC | NM_001001523.1 |
| ILMN_1799467 | SAMD9L | NM_152703.2 |
| ILMN_1701621 | SCO2 | NM_005138.1 |
| ILMN_1701237 | SH2D1B | NM_053282.4 |
| ILMN_2203896 | SMAD7 | NM_005904.2 |
| ILMN_1690262 | SNAI3 | NM_178310.1 |
| ILMN_1691364 | STAT1 | NM_139266.1 |
| ILMN_1690105 | STAT1 | NM_007315.2 |
| ILMN_1668351 | TAF6 | NM_139315.1 |
| ILMN_1674009 | TKTL1 | NM_012253.2 |
| ILMN_2194229 | TMEM128 | NM_032927.2 |
| ILMN_1725387 | TMEM200A | NM_052913.2 |
| ILMN_1727271 | WARS | NM_173701.1 |
| ILMN_2337655 | WARS | NM_004184.3 |
