# Supplementary material for: Systems Biology Approaches Reveal a Specific Interferon-Inducible Signature in HTLV-1 Associated Myelopathy
Source: PLoS Pathog. 2012 Jan 26;8(1):e1002480. doi: 10.1371/journal.ppat.1002480 (PMC3266939; doi:10.1371/journal.ppat.1002480)
Supplement: Table S4 — Class prediction analysis of the 80-gene transcriptional signature. (DOC) [file ppat.1002480.s013.doc]

**Table S4. Class prediction analysis of the 80-gene transcriptional signature.**

|  | **K-nearest neighbour** | | **Support vector machine** | |
| --- | --- | --- | --- | --- |
|  | **Training set** | **Test set** | **Training set** | **Test set** |
| Accuracy | 0.92 | 0.86 | 0.87 | 0.77 |
| Sensitivity | 0.90 | 0.70 | 0.80 | 0.70 |
| Specificity | 0.93 | 0.92 | 0.90 | 0.80 |
| Area under the ROC^a^ curve | 0.92 | 0.81 | 0.85 | 0.75 |

^a^ROC=receiver operating characteristic
