# Supplementary material for: Systems Biology Approaches Reveal a Specific Interferon-Inducible Signature in HTLV-1 Associated Myelopathy
Source: PLoS Pathog. 2012 Jan 26;8(1):e1002480. doi: 10.1371/journal.ppat.1002480 (PMC3266939; doi:10.1371/journal.ppat.1002480)
Supplement: Table S5 — List of probes used in custom Taqman low density array. (DOC) [file ppat.1002480.s014.doc]

**Table S5. List of probes used in custom Taqman low density array.**

| **Gene Symbol** | **Applied Biosystems Taqman probe ID** |
| --- | --- |
| *Genes associated with HAM/TSP* | |
| ANKRD22 | Hs00944015_m1 |
| GBP1 | Hs00977005_m1 |
| GBP5 | Hs00369472_m1 |
| STAT1 | Hs01013996_m1 |
| EPSTI1 | Hs01566789_m1 |
| P2RY14 | Hs00208434_m1 |
| FCGR1B FCGR1C FCGR1A | Hs00417598_m1 |
| WARS | Hs00188259_m1 |
| IL15 | Hs99999039_m1 |
| CHST7 | Hs00219871_m1 |
| RNF165 | Hs00416377_m1 |
|  |  |
| *Controls* |  |
| ACTB | Hs99999903_m1 |
| HMBS | Hs00609296_g1 |
| GNB2L1 | Hs00914568_g1 |
| RPLP0 | Hs99999902_m1 |
| B2M | Hs99999907_m1 |
| 18sRNA | technical standard included on every microfluidic card |
